# Characterization of the Phytochemical Composition and Bioactivities of *Anacyclus maroccanus* Ball. and *Anacyclus radiatus* Loisel Aerial Parts: Preliminary Evidence for the Possible Development of Moroccan Plants

**DOI:** 10.3390/molecules27030692

**Published:** 2022-01-21

**Authors:** Saida Sissi, Silvia Di Giacomo, Claudio Ferrante, Paola Angelini, Alberto Macone, Anna Maria Giusti, Chiara Toniolo, Annabella Vitalone, Aghraz Abdellah, Mustapha Larhsini, Luigi Menghini, Mohammed Markouk, Gabriela Mazzanti, Antonella Di Sotto

**Affiliations:** 1 Laboratory of Agri-Food, Biotechnology and Valorization of Plant Resources, Phytochemistry and Pharmacology of Medicinal Plants Unit, Faculty of Sciences Semlalia, Cadi Ayyad University, Marrakech 40000, Morocco; saida.sissi@ced.uca.ma (S.S.); abdellah.aghraz@gmail.com (A.A.); larhsini@uca.ac.ma (M.L.); markouk@uca.ac.ma (M.M.); 2 Department of Physiology and Pharmacology “V. Erspamer”, Sapienza University of Rome, P.le Aldo Moro 5, 00185 Rome, Italy; silvia.digiacomo@uniroma1.it (S.D.G.); annabella.vitalone@uniroma1.it (A.V.); 3 Department of Pharmacy, Botanic Garden “Giardino dei Semplici”, University “Gabriele d’Annunzio” of Chieti-Pescara, Via dei Vestini 31, 66100 Chieti, Italy; claudio.ferrante@unich.it (C.F.); luigi.menghini@unich.it (L.M.); 4 Department of Chemistry, Biology and Biotechnology, University of Perugia, 06122 Perugia, Italy; paola.angelini@unipg.it; 5 Department of Biochemical Sciences, A. Rossi Fanelli, Sapienza University of Rome, P.le Aldo Moro 5, 00185 Rome, Italy; alberto.macone@uniroma1.it; 6 Department of Experimental Medicine, Sapienza University of Rome, P.le Aldo Moro 5, 00185 Rome, Italy; annamaria.giusti@uniroma1.it; 7 Department of Environmental Biology, Sapienza University of Rome, P.le Aldo Moro 5, 00185 Rome, Italy; chiara.toniolo@uniroma1.it

**Keywords:** phenolics, hypoglycemic activity, α-glucosidase, antiglycation activity, chelating activity, advanced glycation end products, antimicrobial activity

## Abstract

In the present study, the phytochemical composition and bioactivities of *A. maroccanus* (AM) and *A. radiatus* (AR), two ecotypes collected in the Demnate road and Essaouira regions, respectively, were studied to highlight a pharmacological interest and to enable possible pharmaceutical development. To this end, methanolic and ethyl acetate extracts were prepared for each ecotype by fractionation; next, their phytochemical composition was evaluated by spectrophotometric and chromatographic analysis. Moreover, in line with the available evidence for *Anacyclus* spp. and their traditional use, a screening of bioactivities, including antioxidant, hypoglycemic, antiglycative, chelating, and antibacterial activities, was performed. The extracts were characterized by high amounts of polyphenols, tannins, and flavonoids, especially in the methanolic extracts; these samples were also enriched in carotenoids despite a lower chlorophyll content. Chlorogenic acid and rutin were the major identified compounds. The extracts also showed interesting hypoglycemic, antiglycative, and antibacterial properties, although with differences in efficacy and potency. Present results provide more scientific basis to the ethnopharmacological uses of *Anacyclus* spp. and suggest a further interest in AM and AR ecotypes as natural sources of bioactive compounds and/or phytocomplexes for possible pharmaceutical and nutraceutical developments.

## 1. Introduction

**Figure 1:**
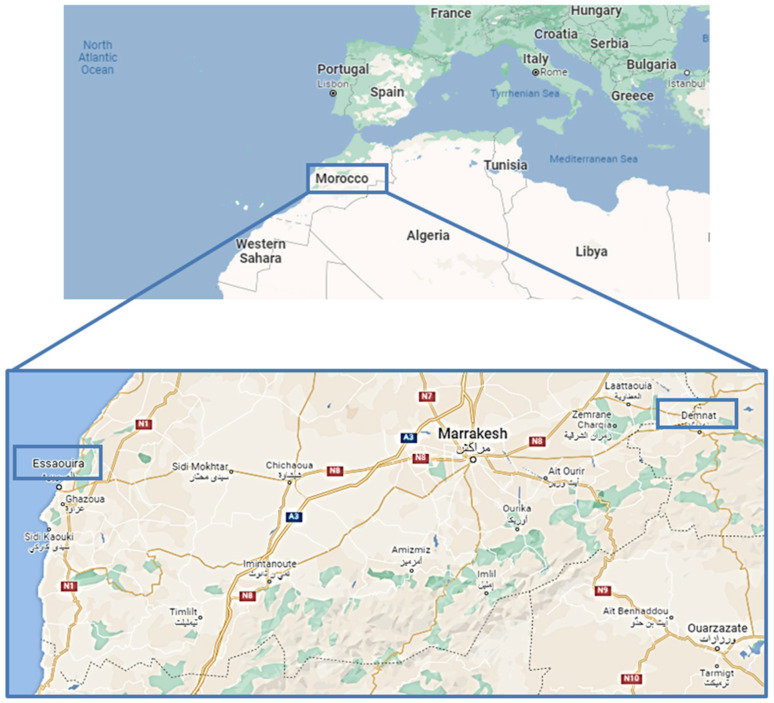
Location of Demnate and Essaouira regions (blue boxes) in the country of Morocco (Source: Google maps).

Morocco is a Mediterranean country characterized by diverse biotopes (desert, mountains, coastal areas, etc.) and a complete range of Mediterranean bioclimates (from humid to Saharan), due to its middle position between two seas (Figure 1) [1,2]. These peculiar ecological conditions provide huge and varied plant biodiversity, among which there are species with potential medical interest [3]. Such species have been classified into 150 families and 940 genera, with around 900 endemic plants [1,2]. These features make Morocco an actual plant genetic reserve and a reservoir of novel bioactive compounds for drug discovery [2]. At the international level, the production of Moroccan medicinal plants and their preparations (such as essential oils or dried extracts) is destined almost entirely for export, with an increased interest in the years due to the opening of novel markets [3].

Moroccan medicinal plants have received a great deal of attention in the field of ethnopharmacology, being exploited as inexpensive and available sources of drugs by the local population, especially in rural areas, for primary health care; knowledge of medicinal plants and their properties are transmitted from generation to generation, being the basis of the old culture of Moroccan people [4]. As highlighted by several ethnobotanical and ethnopharmacological surveys, ancestral medical practices, especially phytotherapy traditions, are well preserved by Moroccan people and are even active today; this widespread use is a result of the accumulation of knowledge from various sources and different ethnic traditions, coupled with the long exposure to, and experience of, these people with natural resources [3]. Medicinal plants have traditionally been exploited by Moroccan people to fight several diseases, such as bacterial and viral infections, gastro-intestinal disorders, diabetes, hypertension, and skin affections, likely due to a richness in phytochemicals, such as polyphenols, saponins, and essential oils, whose bioactivities (e.g., anti-infective, antioxidant, antitumor, and antiviral ones) are known [1,4,5,6]. Moreover, surveys have shown that about 70% of Moroccan people currently use medicinal plants. For example, *Artemisia herba-alba*, *Carum carvi*, and *Nigella sativa* are exploited as antidiabetic and antihypertensive remedies [2], while some species from the Lamiaceae and Asteraceae families are utilized for digestive and genito-urinary disorders [5]; in particular, *Mentha* spp. are used to relieve respiratory and gastro-intestinal disorders [6]. Along with traditional knowledge, a lack of health facilities, scanty accessibility to conventional medicine, and vulnerability of sociocultural status support the rush toward medicinal plants as essential resources for the primary care of poor Moroccan people, especially those living in rural areas [6]. However, the pharmacological basis and phytochemical requirements for the bioactivities remain to be defined. This strengthens the importance of studying Moroccan medicinal plants, not only to valorize their biodiversity and support further developments in pharmaceutical, nutraceutical, and cosmetic fields, but also to give a scientific basis to ethnobotanical traditions, which can lead to their rational and safe use by local people.

Among the endemic plants from Morocco, *Anacyclus* species, belonging to the Asteraceae family, widely occur in this Mediterranean country, with about 12 species distributed in North Africa, South Europe, and the Middle East [7]. These plants are characterized by a close morphological flower similarity, thus being commonly associated with chamomile; indeed, *Anacyclus pyrethrum* DC. is also known as “Spanish chamomile” [8], while *Anacyclus clavatus* (Desf.) Pers. (syn. *Anthemis tomentosa* Gouan) is known as “wooly chamomile” [9]. After all, a common phylogenetic origin of *Anacyclus* and *Matricaria* genera has been highlighted [10]. Many *Anacyclus* species, including *Anacyclus pyrethrum* (L.) Lag., *Anacyclus radiatus* Loisel, *Anacyclus valentinus* L., and *Anacyclus clavatus* (Desf.), have been used traditionally to treat different ailments, such as digestive disorders, pain, and infections, likely due to their antioxidant, anti-inflammatory, analgesic, and antimicrobial properties [11,12,13,14]. In particular, extracts from *A. pyrethrum* root are the most studied, and their benefits have been associated to the presence of alkaloids (e.g., pyrethrine), fatty acids, and unsaturated amides (e.g., pellitorine, anacycline, and sesamin), along with polyphenolic compounds; they have been recommended for treating toothache, digestive problems, infertility, and used as an aphrodisiac, analgesic, nervous system tonic, anti-arthritic, and eupeptic remedies [15,16,17,18]. Furthermore, the aerial parts of *A. pyrethrum* were found to be endowed with antimicrobial and antioxidant properties, likely ascribable to the presence of polyphenolic compounds [13]; similarly, polyphenols, tannins, and flavonoids seem to be responsible for the antioxidant, antimicrobial, and antinflammatory properties of *Anacyclus clavatus* (Desf.) aerial parts, which are recognized to possess multiple beneficial properties [19,20]. Finally, the essential oil from aerial parts of *Anacyclus valentinus* L. has shown antifungal properties [21].

**Figure 2:**
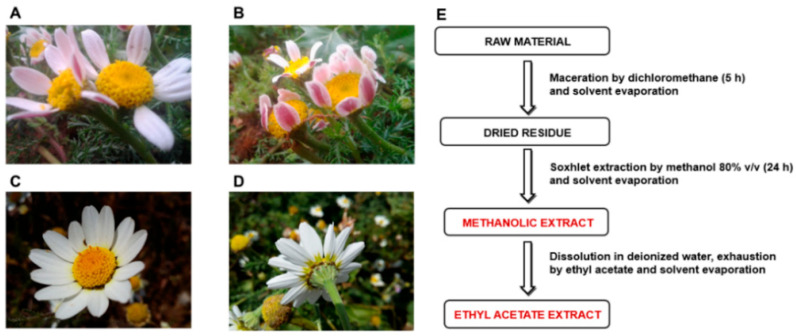
Inflorescences of *Anacyclus maroccanus* Ball (**A**,**B**) and *Anacyclus radiatus* Loisel ecotypes (**C**,**D**) from the Demnate and Essaouira regions of Morocco, respectively. (**E**) Extraction procedures used to obtain the methanolic and ethyl acetate extracts of Moroccan *Anacyclus* ecotypes.

In the present study, *Anacyclus maroccanus* Ball (AR; Figure 2A,B) and *Anacyclus radiatus* Loisel (AR; Figure 2C,D) ecotypes, collected in the Demnate and Essaouira regions of Morocco, respectively (Figure 1), were studied for their phytochemical composition and bioactivities to valorize their biodiversity and highlight any possible future pharmacological interests. While *A. maroccanus* is not mentioned in traditional medicine, infusions of *A. radiatus* flowers are used to treat stomach upsets and microbial infections [22].

To perform the study, methanolic and ethyl acetate extracts from flowering aerial parts of each ecotype were prepared by fractionation (Figure 1E); next, the polyphenolic composition was evaluated by a multimethodological spectrophotometric and chromatographic analysis. We focused on the flowering aerial parts, which are rich sources of polyphenols and recognized to possess multiple beneficial properties, making them interesting candidates for pharmaceutical and nutraceutical applications. In particular, the dietary polyphenols showed antidiabetic effects, likely due to their ability to affect carbohydrate metabolism, and cytoprotection against hypoglycemia-induced oxidative stress [23].

In line with this evidence, the ability of the extracts to affect the enzymes involved in carbohydrate metabolism and hyperglycemia-associated stress (oxidative stress, formation of advanced glycation end products) have been evaluated. In particular, the antioxidant power and carbohydrate enzyme inhibition, along with the antiglycative and chelating properties, have been studied. In addition, considering the traditional use of AR aerial parts, the antimicrobial properties of the extracts against different bacterial, fungal, and dermatophytic species, along with a bioinformatic evaluation of the possible interactions accounting for antimicrobial activity, have also been assessed.

## 2. Results

### 2.1. Phytochemical Analysis

#### 2.1.1. Spectrophotometric Analysis

High levels of total polyphenols, tannins, and flavonoids were found in all of the tested extracts, especially in those obtained from the inflorescences of *A. radiatus* (Table 1). Moreover, their levels were higher in methanolic extracts than in ethyl acetate extracts (Table 1). Indeed, about double the amounts of total polyphenols and tannins were measured in the AM (*A. maroccanus*) methanolic extract with respect to the ethyl acetate extract. Similarly, the tannin content was doubled in the AR (*A. radiatus*) methanolic extract with respect to the ethyl acetate extract, while the total polyphenols were almost tripled.

Regarding flavonoids, the AM methanolic extract contained triple the level with respect to the ethyl acetate fraction; by contrast, a slight 1.5-fold increase was found in the respective AR extract. Furthermore, despite similar flavonoid amounts in ethyl acetate extracts, the AM methanolic extract contained double the level of flavonoids compared to the AR sample.

For both species, the content of total chlorophylls was higher in the ethyl acetate extracts with respect to the methanolic extracts, being about doubled in the AM sample; conversely, similar levels were detected in the methanolic extracts (Table 2). Both chlorophyll A (Chl A) and B (Chl B) were significantly higher in the AM ethyl acetate extract with respect to the AR extract, with the amount being about doubled; however, Chl B was the most representative compound. Methanolic extracts contained similar levels of Chl A, while Chl B was slightly higher in the AR sample (Table 2). Carotenoids were mainly concentrated by methanol, achieving a maximum amount in the AR sample, which was about 2.5-fold higher than that of the AM sample. Similarly, carotenoid amount in the AR ethyl acetate extract was about 1.5-fold higher than that in the AM one. The opposite trend in the content of chlorophylls and carotenoids in the AM and AR extracts was also confirmed by the chlorophyll and carotenoid ratio, which was higher than 1 for all of the samples, except for the AR methanolic extract, likely due to the high amount in Chl B (Table 2).

#### 2.1.2. Chromatographic Analysis of Phenolic Compounds

**Figure 3:**
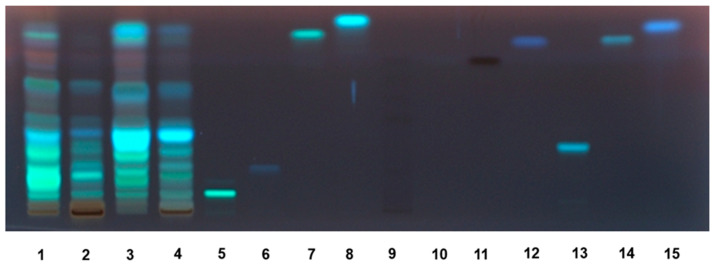
HPTLC chromatogram of *Anacyclus maroccanus* Ball (AM) and *Anacyclus radiatus* Loisel (AR) dry extracts under UV 366 nm after Anisaldehyde/Natural Product Reagent (NPR) derivatization. 1. AM ethyl acetate extract; 2. AM methanolic extract; 3. AR ethyl acetate extract; 4. AR methanolic extract; 5. rutin; 6. chlorogenic acid; 7. quercetin; 8. kampferol; 9. epigallocatehin; 10. gallic acid; 11 catechin; 12. caffeic acid; 13. luteolin 7-O-glucoside; 14. luteolin; 15. apigenin.

The high-performance thin-layer chromatographic (HPTLC) analysis highlighted the presence of different phenolic compounds in the AM and AR extracts, which have been visualized under different wavelengths by means of suitable derivatization reagents (Figure S1). As displayed by the HPTLC chromatogram of the extracts under UV 366 nm after Anisaldehyde and Natural Product Reagent derivatization (Figure 3), among the eleven standard polyphenols, rutin (Retention factor, Rf 0.09), chlorogenic acid (Rf 0.20), and quercetin (Rf 0.78) were identified in all of the extracts. According to the results of HPLC analysis (Figure S2, Table 3), rutin was found to be the most abundant flavonoid in the ethyl acetate extracts from both species, especially in AR samples. The amounts in the AM and AR ethyl acetate extracts were about 11- and 28-fold higher than those found in the respective methanolic samples (Table 3). Similarly, almost 14- and 6-fold higher amounts of quercetin were measured in the AM and AR ethyl acetate extracts with respect to the methanolic extracts. Conversely, chlorogenic acid showed 1.2- to 1.4-fold increased levels in methanolic samples compared to the ethyl acetate ones; moreover, it was slightly more concentrated in the AR extracts with respect to AM ones. Among the other identified compounds, AR extracts were enriched in benzoic acid (content about 8- to 10-fold higher than that of AM samples), especially in the ethyl acetate one (content at least 4-fold higher than that of the methanolic extract). Accordingly, caffeic acid, ferulic acid, gallic acid, syringic acid, and syringaldehyde were more abundant in the AM ethyl acetate extracts, with about 2- to 4-fold higher amounts than that in the respective AM samples. Traces of coumaric acid were found in the AR ethyl acetate extract. AM and AR ethyl acetate extracts also contained high levels of epicatechin, which was doubled in the AR sample with respect to the AM one. Moreover, the AR sample contained almost a 7-fold higher amount of catechin than the respective methanolic sample; conversely, AM extracts displayed similar levels of catechin, with about 5-fold lower levels between the AM and AR ethyl acetate extracts, and a slight 1.4-fold difference in the respective methanolic samples.

#### 2.1.3. Gas Chromatography–Mass Spectrometry (GC–MS) Analysis

Forty-one compounds belonging to different classes of natural compounds, among which dicarboxylic acids, alpha-hydroxy acids, phenolic acids (both hydroxycinnamic and hydroxybenzoic derivatives), and fatty acids were identified using GC-MS analysis in all the tested extracts (Figure S3). Among dicarboxylic acids, meglutol and methylmalonic acid were especially abundant in the methanolic extracts of both species, while azelaic acid and butanedioic acid were abundant in the ethyl acetate extracts; moreover, caffeic acid, coumaric acid, isovanillic acid, protocatechuic acid, and salicylic acid were the main phenolic acids highlighted in ethyl acetate extracts. Interestingly, ferulic acid was highly concentrated in the AM methanolic sample, despite a lower abundance in the other extracts. Methanolic extracts also better concentrated glycerol and L-pyroglutamic acid. More details about the identified compounds and their relative abundance in the extracts are displayed in Table 4.

### 2.2. Screening of Biological Activities

Different possible bioactivities of AM and AR extracts were evaluated in order to highlight a potential industrial interest. In particular, both direct and indirect antioxidant mechanisms were studied. Radical scavenger power against synthetic DPPH and ABTS radicals was assessed as a direct antioxidant mechanism; moreover, the ferrous chelating power was studied as a kind of indirect antioxidant activity. Indeed, metal chelating ability lowers ferrous ion availability, which is required in the Fenton reactions, leading to a reduction in the formation of reactive oxygen species.

#### 2.2.1. Antioxidant Activities

**Figure 4:**
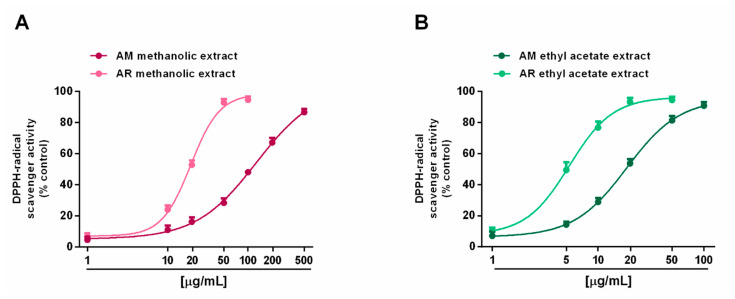
Scavenger activity of *Anacyclus maroccanus* Ball (AM) and *Anacyclus radiatus* Loisel (AR) dry extracts against the DPPH^·^ radical. (**A**) Methanolic extracts. (**B**) Ethyl acetate extracts. Data represents the average and standard error of at least six replicates from at least two experiments.

**Figure 5:**
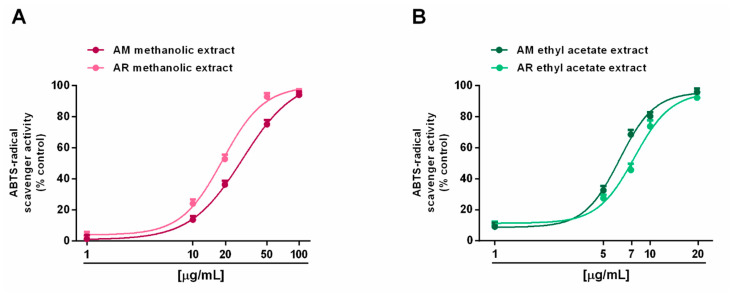
Scavenger activity of *Anacyclus maroccanus* Ball (AM) and *Anacyclus radiatus* Loisel (AR) dry extracts against the ABTS radical. (**A**) Methanolic extracts. (**B**) Ethyl acetate extracts. Data represents the average and standard error of at least six replicates from at least two experiments.

Radical scavenging ability was evaluated towards DPPH and ABTS radicals in the concentration range of 1 to 500 μg/mL. Under our experimental conditions, all the extracts exhibited interesting radical scavenging activities, although with different potency and efficacy (Figure 4 and Figure 5). When assayed against the DPPH radical, samples from AR were more effective than those from AM; moreover, ethyl acetate extracts were more potent than the methanolic extracts (Figure 4A,B). In particular, the AR methanolic extract completely scavenged the radical at a concentration of 50 μg/mL (about 93% DPPH inhibition), while a 10-fold higher concentration was required to achieve the maximum scavenging effect for the AM methanolic extract (about 87% DPPH inhibition). Comparing the samples from the same species, the AM ethyl acetate extract produced a maximum 91% DPPH-inhibition at a concentration of 100 μg/mL, at which a half effect was produced by the AM methanolic extract. Similarly, 10 μg/mL of the AR ethyl acetate extract inhibited DPPH by about 77%, despite a 25% inhibition by the AR methanolic sample (Figure 4A,B). These different potencies were confirmed by the IC_50_ values (Table 5); indeed, values for the AM and AR ethyl acetate extracts were almost 7-fold and 4-fold lower than those of the respective methanolic samples. Similarly, the AM methanolic and ethyl acetate extracts were about 7- and 4-fold less potent than their respective AR samples. Regarding the ABTS assay (Figure 5A,B), all of the extracts were able to fully scavenge the ABTS radical, with the ethyl acetate extracts being the most potent samples, especially that from the AM species. Conversely, the AR methanolic extract was more potent than that from the AM species. The IC_50_ values confirmed these behaviors (Table 5), with that of the AM ethyl acetate extract being about 1.3-fold lower than the value for the AR sample, while an opposite trend was highlighted for the methanolic extracts (about 1.4-fold higher potency for the AR sample). As expected, the positive control trolox was usually more potent than the extracts of both species, except for the AR ethyl acetate extract, which exhibited a similar and slightly lower (almost 1.7-fold lower with respect to trolox) IC_50_ value than in the DPPH assay (Table 5).

#### 2.2.2. Inhibition of Advanced Glycation End-Product (AGE)

**Figure 6:**
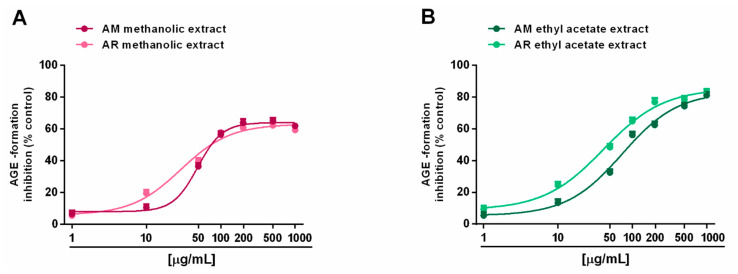
Ability of *Anacyclus* *maroccanus* Ball (AM) and *Anacyclus radiatus* Loisel (AR) dry extracts to inhibit the formation of advanced glycation end products (AGEs). (**A**) Methanolic extracts. (**B**) Ethyl acetate extracts. Data represents the average and standard error of at least six replicates from at least two experiments.

Regarding the formation of advanced glycation end products (AGEs), both the AM and AR ethyl acetate extracts were able to block AGE generation, despite only a partial effect (maximum 60% inhibition) by methanolic samples (Figure 6A,B). According to the results of the antioxidant activity assays, IC_50_ values showed that the AR ethyl acetate extract was almost two-fold more potent that the respective AM extract (Table 6). By contrast, the IC_50_ values of the methanolic extracts were not evaluated, being that the highest achieved an effect lower than 80%. As expected, the positive control rutin was almost 15- to 25-fold more potent than the AR and AM ethyl acetate extracts, respectively (Table 6).

#### 2.2.3. Iron Chelating Activity

**Figure 7:**
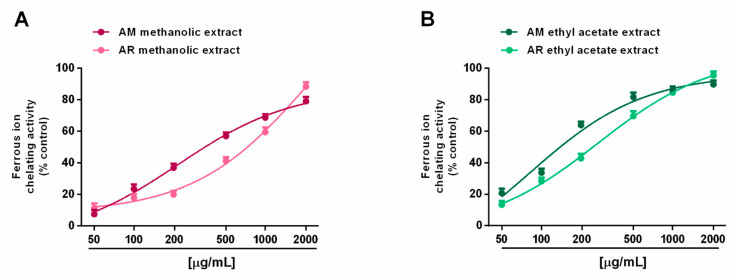
Ability of *Anacyclus maroccanus* Ball (AM) and *Anacyclus radiatus* Loisel (AR) dry extracts to chelate ferrous ions in the ferrozine assay. (**A**) Methanolic extracts. (**B**) Ethyl acetate extracts. Data represents the average and standard error of at least six replicates from at least two experiments.

In the ferrozine assay, all of the tested extracts were able to chelate ferrous ions in a concentration range of 50 to 2000 μg/mL, with a great potency of ethyl acetate samples (Figure 7A,B). Surprisingly, both AM extracts were more potent than their respective samples from the AR species. Indeed, at a concentration of 200 μg/mL, the AM methanolic and ethyl acetate extracts produced a chelating effect of about 40% and 60%, respectively, despite a 20% and 40% inhibition of the respective AR samples. This behavior was confirmed by the IC_50_ values, which were about doubled in the AR samples with respect to the AM ones; interestingly, both the AM and AR ethyl acetate extracts, and the AM methanolic extract, showed at least half IC_50_ values with respect to the positive control rutin (Table 6).

#### 2.2.4. In Vitro Metabolic Enzyme Inhibition

**Figure 8:**
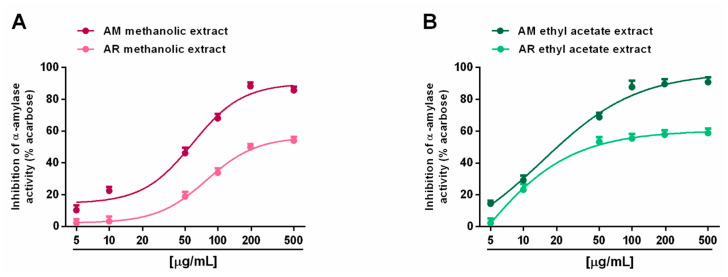
Ability of *Anacyclus maroccanus* Ball (AM) and *Anacyclus radiatus* Loisel (AR) dry extracts to inhibit the α-amylase enzyme. (**A**) Methanolic extracts. (**B**) Ethyl acetate extracts. Data represent the average and standard error of at least six replicates from at least two experiments.

**Figure 9:**
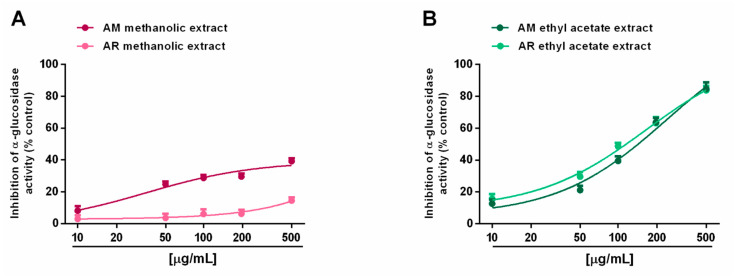
Ability of *Anacyclus maroccanus* Ball (AM) and *Anacyclus radiatus* Loisel (AR) dry extracts to inhibit the α-glucosidase enzyme. (**A**) Methanolic extracts. (**B**) Ethyl acetate extracts. Data represent the average and standard error of at least six replicates from at least two experiments.

When assayed for metabolic enzyme inhibition, all of the extracts (5–500 µg/mL; dilution factor 1:2 or 1:2.5) were able to progressively reduce the α-amylase activity in a concentration-dependent manner, with the AM methanolic and ethyl acetate extracts being the most effective (Figure 8A,B). Indeed, despite the complete inhibition of metabolic enzymes induced by these samples, the AR extracts produced a maximum 60% effect, thus hindering the evaluation of the IC_50_ values. Comparing the AM samples, 50 µg/mL of ethyl acetate extract hindered α-amylase by about 70%, despite a 45% inhibition by the methanolic sample (Figure 8A,B). The IC_50_ values showed that the AM ethyl acetate extract was at least 3-fold more potent than the methanolic sample, and at least 5-fold more potent than the positive control acarbose (Table 7). In the α-glucosidase inhibition assay, the methanolic extracts of both *Anacyclus* species produced a weak enzyme inhibition (lower than 40%); conversely, the ethyl acetate extracts exhibited similar inhibitory power, with the AM sample being slightly more potent (Figure 9A,B). Indeed, its IC_50_ value was about 1.7-fold lower than that of the AR extract; however, both samples were at least 100-fold less potent than acarbose (Table 7).

The prominent phenolic compound found in the ethyl acetate extracts was rutin; in this regard, through a docking approach, we calculated the putative affinity of rutin towards α-glucosidase (Ki = 29 nM) and α-amylase (Ki = 180 nM) (Figure S4). The putative affinity of rutin towards the enzyme is consistent with the micromolar concentrations (0.8–8 µM) of rutin in the AM and AR ethyl acetate extracts, at their respective IC_50_ values towards both enzymes (Table 7). Additionally, the rutin Ki values are also very distant from the putative LC_50_ value (1.6 mM; 72.31 mg/mL) towards the *Daphnia magna* toxicological model as calculated through the Toxicity Estimation Software Tool (T.E.S.T.), thus suggesting a good biocompatibility of the extracts, which supports the rationale for their pharmacological use. However, the low bioavailability of rutin predicted by the SwissADME platform could limit their use at local sites, such as the gastrointestinal tract, for the inhibition of α-glucosidase and α-amylase, or the skin and mucous membranes for treating infectious diseases, as indicated by the in vitro antimicrobial results described below.

#### 2.2.5. Antimicrobial Activity

**Figure 10:**
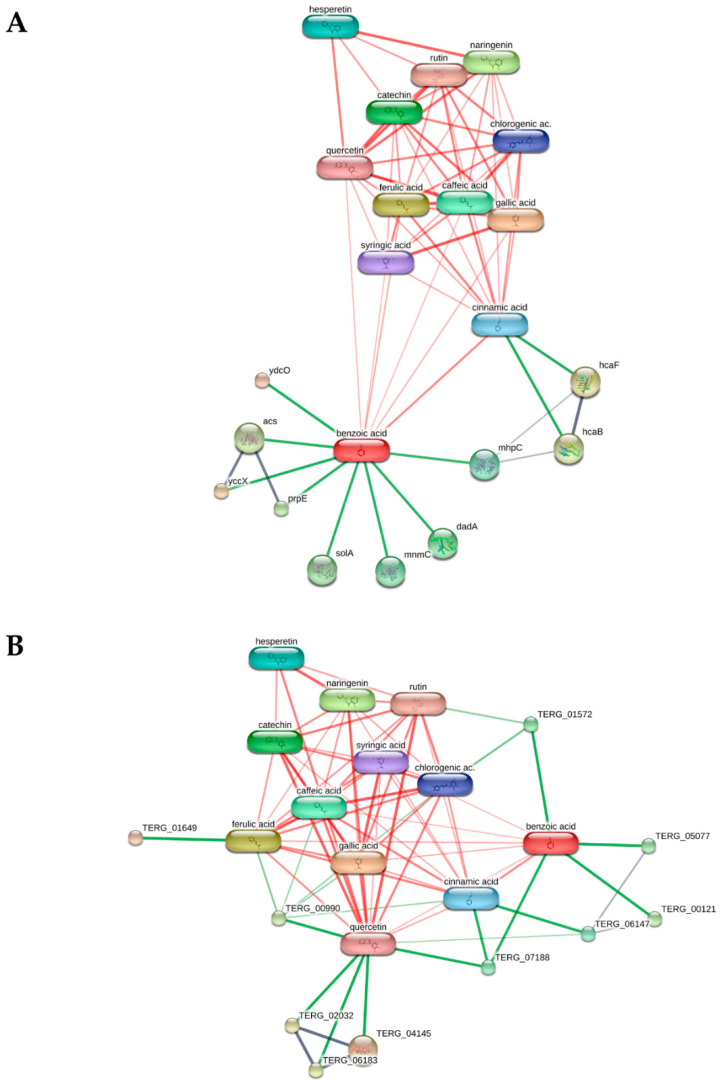
Components-targets analysis highlighting the putative interactions between the extracts’ phytochemicals and bacterial proteins. (**A**) The KEGG analysis conducted on *E. coli* via the platform STITCH showed the potential influence of benzoic acid and coumaric acid on the bacterial pathways KEGG00360 (phenylalanine metabolism) and KEGG01220 (degradation of aromatic compounds). (**B**) The components-targets analysis conducted on *T. rubrum* showed putative interactions of quercetin, cinnamic acid, and benzoic with different dermatophytes enzymes, without identifying any discrete metabolic pathway affected by the aforementioned phytochemicals.

The methanolic and ethyl acetate extracts from *Anacyclus maroccanus* Ball (AM) and *Anacyclus radiatus* Loisel (AR) were tested against different bacterial, fungal, and dermatophytes species (Table 8, Table 9 and Table 10). Specifically, among all tested strains, *E. coli* and *T. rubrum* were more sensitive to the growth inhibitory effects induced by the extracts. With the aim of further unravelling the putative mechanisms underlying the inhibition of *E. coli* and *T. rubrum* growth, a bioinformatics analysis was conducted on the platform STITCH (Figure 10A) suggested the influence of the extracts on the phenylalanine metabolism (KEGG00360) and degradation of aromatic compounds (KEGG01220) of *E. coli*. In the scenario of the putative interactions shown by the components-targets analysis, a prominent position was played by benzoic acid and cinnamic acid, which were reputed to interact with the following enzymes involved in the aforementioned metabolic pathways: 3-phenylpropionate dioxygenase subunit beta (hcaF), 2,3-dihydroxy-2,3-dihydrophenylpropionate dehydrogenase (hcaB), alpha/beta hydrolase fold protein (mhpC), and D-amino acid dehydrogenase small subunit (dadA). Regarding *T. rubrum*, the component-target analysis reported in Figure 10B showed the prominent position of benzoic acid, cinnamic acid, and quercetin; different dermatophytes proteins involved in the microbial metabolism were predicted to interact with these phenolic compounds (Figure 10B). However, the analysis conducted on *T. rubrum* did not permit the identification of the putative metabolic pathways affected by the phytochemicals.

## 3. Discussion

In the present study, methanolic and ethyl acetate extracts from the flowers of *Anacyclus maroccanus* Ball and *Anacyclus radiatus* Loisel, two Moroccan ecotypes, have been characterized for their polyphenolic composition and for the polyphenol-associated bioactivities, especially the ability to affect the enzymes involved in carbohydrate metabolism and hyperglycemia-associated stress (oxidative stress, formation of advanced glycation end products), through antioxidant, antiglycative, and chelating properties. In line with the traditional use of AR aerial part infuses, the antimicrobial activity of the extracts has been evaluated too.

*Anacyclus* species are widely exploited in folk medicine of North Africa and other Mediterranean countries, due to their recognized therapeutic properties ascribed to the presence of different compounds, including flavonoids, terpenoids, and alkaloids [7]. Although *A. pyrethrum* DC. is the most studied variety, other endemic species are used as remedies for primary care by local people, who have limited access to conventional medicine [19,20,21]; however, the mechanisms accounting for its bioactivities, along with the composition of bioactive compounds, remain to be defined. This strengthens the need to promote pharmacognostic research on these species in order to not only to valorize their biodiversity by highlighting their relevance as sources for drug discovery and industrial fields, but also to support their traditional use through the clarification of the pharmacological basis and the phytochemical features required to achieve the expected effect. This point is also relevant to limit the problem of the invalid metabolic panaceas (IMPs), which often occurs due to the poorly characterized biological activity profile of extracts or pure compounds [24].

Concerning the extracts from *A. maroccanus* and *A. radiatus*, a phytochemical analysis revealed that methanolic extracts from both species, especially that from AM, were richer in polyphenols, tannins, flavonoids, and carotenoids than the ethyl acetate extracts; conversely, the ethyl acetate extracts contained higher amounts of total chlorophylls. Although the AM and AR species have been scantly investigated, the results obtained in this study agree with the composition of aerial parts of other *Anacyclus* species. Indeed, Selles et al. [13] reported that a methanolic extract from the aerial parts of *A. pyrethrum* contained the highest amount in total polyphenols with respect to the aqueous and chloroform extracts; likewise, a methanolic extract from *A. clavatus* was richer in total polyphenols than the aqueous one [17]. The tannin content of the AM methanolic extract was similar to that of a methanolic extract from *A. clavatus* aerial parts (39.21 mg TAE/g) [13], while the amount from the respective AR sample was higher by about 1.4-fold. Regarding flavonoids, the methanolic extracts from the AM and AR samples contained levels about 1.5- and 3-fold lower than that of *A. pyrethrum* aerial parts (92.50 mg QE/g), respectively [13]. Moreover, the amount in the AM methanolic extract was almost doubled with respect to that of *A. clavatus* (9.96 mg QE/g)*,* despite a very similar level in the AR sample [17]. These data highlight that our AM and AR species, albeit producing slight differences due to the species, cultivation condition, habitat, and extraction method, represent interesting sources of polyphenols, just like the most studied *Anacyclus* species.

The polyphenol composition of *Anacyclus* spp. has not been thoroughly investigated. According to the compounds identified in the AM and AR methanolic samples, Bouriche et al. [17] highlighted the presence of gentisic acid (2,5-dihydroxybenzoic acid), chlorogenic acid, 4-hydroxybenzoic acid, protocatechuic acid, caffeic acid, vanillic acid, and rutin in the methanolic extract from *A. clavatus* aerial parts. Of note, compounds never before identified in these species, to the best of our knowledge, are meglutol, epicatechin, and ferulic acid. Altogether, these data constitute the first complete phytochemical characterization of AM and AR species and suggest a possible involvement of identified compounds in the bioactivities of the extracts, which deserves further study.

When assayed for antioxidant activity, all of the samples displayed a similar efficacy towards the ABTS radical, while methanolic and ethyl acetate AR extracts were found to be more potent than their respective AM extracts against the DPPH radical. In line with our data, Selles et al. [13] reported that a methanolic extract from *A. pyrethrum* aerial parts was more potent than water and chloroform extracts in scavenging DPPH, with an IC_50_ value (56 μg/mL) comparable to that of ascorbic acid (48 μg/mL), which was used as positive control. Likewise, the methanolic extract from *A. clavatus* strongly inhibited DPPH, with a IC_50_ value of 28.30. Based on our results, the presence of DPPH-radical scavenger compounds in all the extracts from the AM and AR species can be hypothesized; moreover, fractionation by ethyl acetate seems to concentrate the bioactive compounds, thus leading to an IC_50_ lower than that of the methanolic extract and of the positive control trolox.

DPPH and ABTS are two synthetic radicals that can be scavenged by electron- or hydrogen-transfer mechanisms, and that have different specificities and kinetics: DPPH is more selective for small molecules, being limited sterically with regard to access to the radical site, while ABTS reacts with both lipophilic and hydrophilic compounds with a poor selectivity in the reaction with hydrogen-atom donors [25]. Indeed, ABTS is bleached by different antioxidants in fruits and vegetables while DPPH seems to not react with carotenoids [25].

Based on this evidence, the ABTS-scavenging abilities of AM and AR extracts could be ascribable to the presence of both hydrophilic and lipophilic phytochemicals, such as polyphenols, tannins, and carotenoids; conversely, the higher potency of AR samples suggests the presence of specific, or more concentrated, scavenging compounds, such as phenolic acids and flavonoids. However, further studies are required to clarify their involvement in the observed radical scavenging activity.

The extracts have also been evaluated for their ability to chelate ferrous ions and to inhibit the production of advance glycation end-products (AGEs), which are toxic metabolites accumulated under different pathologies, and are responsible for inflammation and oxidative stress [26]. Along with AGE, alterations in iron homeostasis are typical of diabetes, and have been found to be associated with an increased production of reactive oxygen species (ROS) and hyperglycemia complications [27]; therefore, the metal chelating ability and AGE inhibition can provide benefits in hyperglycemia conditions, blocking both oxidative and inflammatory damage.

In the present study, the AM and AR ethyl acetate extracts showed the most effective antiglycative capacity and the most potent chelating activity. Evidence from the literature has highlighted the ability of different phenolic compounds, among which are rutin and catechin, to exert similar properties; moreover, gallic acid and catechin prevent AGE formation by trapping α-dicarbonyl compounds or by inhibiting the formation of Amadori products [28,29]. The presence of these compounds in the tested extracts can support our hypothesis about their involvement in the extract bioactivities.

In line with the evidence of the antiglycative and chelating properties, in order to better disclose the possible interest in the tested extract in the management of glucidic metabolism disfunction, such as hyperglycemia and diabetes, their ability to interfere with the activity of key enzymes involved in carbohydrate metabolism to glucose, i.e., α-amylase and α-glucosidase, has been studied. In particular, α-amylase is a salivary and pancreatic enzyme that catalyzes the endo-hydrolysis of α-1,4-glucosidic linkages of amylose, while α-glucosidases further breaks down α-1,4-glucosidic linkages in the small intestine, allowing glucose release and absorption [30].

Under our experimental conditions, both methanolic and ethyl acetate extracts from AM species significantly inhibited α-amylase, despite a weak effect of the AR samples. Conversely, α-glucosidase inhibition was mainly induced by ethyl acetate extracts, which agrees with their chelating and antiglycative properties. Despite the differences in the ability of the tested extracts to mainly affect glycolytic enzyme activity, present data agree with previous evidence about the ability of extracts from *A. pyrethrum* root and *A. valentinus* aerial parts to counteract hyperglycemia in in vivo models of diabetes [14,31]; however, the bioactive compounds, and their true mechanisms of action, remain to be elucidated.

Multiple sources of evidence have highlighted a role of dietary phenolics in the regulation of carbohydrate metabolism, particularly as inhibitors of α-amylase and α-glucosidase enzymes [30]. Among them, the flavonoid rutin has been reported to possess antiglycative properties and to deactivate α-amylase and α-glucosidase by forming an inactive complex [32,33]. In the ethyl acetate extracts, rutin is the prominent phenolic compound, and the in silico assessment indicated putative submicromolar affinities of rutin towards both α-amylase and α-glucosidase; this further indicates that this phytochemical plays a key role in the enzyme inhibition by the ethyl acetate extracts.

Moreover, phenolic acids, such as gallic acid and ferulic acid, are known to inhibit the glycolytic α-amylase and α-glucosidase enzymes [34,35]. Compounds containing more than one hydroxyl group, such as caffeic and protocatechuic acids, potently inhibit α-glucosidase [33,34]; conversely, chlorogenic acid seems to mainly block α-amylase [36]. In silico studies have also highlighted the α-glucosidase inhibitory properties of catechin [37]. Further attention can be devoted to meglutol or 3-hydroxy-3-methyl glutaric acid, which is known to induce hypoglycemia and to regulate lipid metabolism [38,39].

Finally, the extracts were found to possess antimicrobial properties, being able to counteract the growth of different bacteria, fungi, and dermatophytes. Both AM and AR ethyl acetate extracts were effective against *E. coli* and *T. rubrum* strains, with a lower potency of methanolic extracts against *E. coli*, especially that from AR, being observed. This last sample also showed a moderate inhibition against *Bacillus cereus*, *Pseudomonas aeruginosa*, *Staphylococcus aureus*, and *Candida albicans*, despite a weak activity for the other samples. The antimicrobial properties of AM and AR samples agrees with the results of Selles et al. [13], which found that a methanolic extract from *A. pyrethrum* aerial parts was able to inhibit *S. aureus* and *B. cereus*, despite slightly affecting *E. coli* and *Pseudomonas aeruginosa*; however, the tested extracts showed lower potency, in terms of MIC values (µg/mL), compared to the reference drugs ciprofloxacin, fluconazole, and griseofulvin for the antibacterial and antifungal effects [40]. In fact, the MIC values of ciprofloxacin, fluconazole, and griseofulvin were always ≤4 µg/mL towards the tested bacterial and fungal strains [40]. We have to consider that the extracts’ MIC values for the ethyl acetate samples were of the same order compared to the reference drugs, with values < 10 µg/mL, with regard to growth inhibitory effects against *E. coli* and *T. rubrum* species. Additionally, considering the content in polyphenolic compounds of the extracts, and according to what reported in previous studies [41], we could hypothesize that these phytochemicals could influence, albeit in part, the observed bacteriostatic and mycostatic effects. Present data are also consistent with the antimicrobial properties of herbal extracts containing benzoic acid, cinnamic acid, and quercetin [42,43,44,45], and collectively suggest a rationale for the use of *Anacyclus* extracts as sources of biomolecules with antimicrobial effects.

Despite this evidence, the true bioactive compounds of *Anacyclus maroccanus* and *Anacyclus radiatus* remain to be clarified and the possible involvement of the tangled interactions among these phytochemicals cannot be excluded.

## 4. Materials and Methods

### 4.1. Chemicals and Reagents

All chemicals, including Folin-Ciocalteu’s phenol reagent, tannic acid (Ph Eur purity), aluminium chloride hexahydrate (AlCl_3_ × 6 H_2_O; Ph Eur purity), 1,1-diphenyl-2-picrylhydrazyl radical (DPPH; 95% purity), 2,2′-azino-bis (3-thylbenzothiazoline-6-sulfonic acid) diammonium salt (ABTS; 98% purity), 2,2′-azobis (2-methylpropionamidine) dihydrochloride (AAPH; 97% purity), ferrozine (97% purity), hydroxylamine hydrochloride (98% purity), iron (III) chloride (FeCl_3_ × 6H_2_O; 97% purity), iron (II) sulfate heptahydrate (FeSO_4_ × 7H_2_O; 99% purity), trolox (97% purity), quercetin (98% purity), rutin (99% purity), tannic acid (98% purity), bovine serum albumin, glucose, fructose, sodium azide, 4-nitrophenyl β-D-glucopyranoside (PNPG), acarbose, and the analytical-grade solvents ethyl acetate (AcOEt; 99.8% purity), iron (II) chloride (FeCl_2_ × 4H_2_O; 99% purity), polyvinylpyrrolidone (PVP), potato starch, sodium carbonate, the enzymes α-amylase from a hog pancreas (50 U/mg), and α-glucosidase from *Saccharomyces cerevisiae* (≥10 U/mg protein), as well as the analytical grade solvents were purchased from Merck (Darmstadt, Germany).

### 4.2. Plant Collection and Identification

Inflorescences of *Anacyclus maroccanus* Ball and *Anacyclus radiatus* Loisel were collected respectively in the Demnate road (Alt. 31°39′22″ and Lon. N 7°25′28″ W) and Essaouira (Alt. 31°29′13″ N and Lon. 9°45′44.8″ W) regions of Morocco. The species were identified by Prof. Ouhammou Mohamed and a voucher specimen for each plant was deposited at MARK-Herbarium of Faculty of Science Semlalia Cady Ayyad University (Marrakech, Morocco). Before processing, defect-free flowers were selected, and all the soil residues were removed. The plant material was dried and stored in a dark and dry environment until use.

### 4.3. Extraction Procedures

For each *Anacyclus* sample, the flowering aerial parts were dried and then subjected to multiple extraction steps to obtain different fractions enriched in phenolic compounds (Figure 2E). Preliminarily, the powdered raw material (150 g) has been left under maceration in dichloromethane (800 mL) for 5 h to dissolve chlorophylls. The extraction residue was thus recovered, dried at room temperature (25 °C) for 2 h, and further extracted in 80% methanol by Soxhlet for 24 h. The obtained methanolic extract was filtered and dried under vacuum (at 45 °C). In order to prepare the ethyl acetate extracts, the methanolic extract was dissolved in distillated water and exhausted by ethyl acetate at least 5 times. Finally, the obtained residue was evaporated at 45 °C by means of a rotavapor. All the extracts were stored at 4 °C before the analysis.

### 4.4. Phytochemical Analysis

#### 4.4.1. Determination of Total Polyphenols, Tannins, and Flavonoids

The total amount of polyphenols, tannins, and flavonoids was determined using the Folin-Ciocalteu and the aluminium chloride spectrophotometric methods, described by Di Sotto et al. with minor changes [46]. For both polyphenols and tannins, the absorbance was measured at 765 nm and the amount was expressed as tannic acid equivalents (TAEs), while total flavonoids were measured at 415 nm and expressed as quercetin equivalents (QE) per gram of sample. Equations of calibration curves for tannic acid and quercetin, calculated by linear regression (GraphPad Prism™ 6.00), were Y = 0.001751X + 0.1101 (r^2^ = 0.986) and Y = 0.003059X + 0.1661 (r^2^ = 0.976), respectively.

#### 4.4.2. Determination of Total Carotenoids and Chlorophylls

The analysis was performed according to previous methods [44]. Briefly, the extracts were dissolved in DMSO, then magnesium oxide (MgO; 100 mg) was added to prevent chlorophyll pheophytinization; thereafter, the mixture was used for determining the chlorophyll a and b content, as well as total carotenoids, by using the absorption coefficient reported by Wellburn [47]. Absorption spectra of the chloroform phase were recorded by a Jenway 6400 spectrophotometer at the wavelengths 480, 649, 665, and 760 nm. The content of chlorophylls a and b, as well as that of the total carotenoids (mg/mL), has been determined by applying the equations chlorophyll A = 11.47 A_665.6_ − 2A_647.6_; chlorophyll B = 21.85 A_647.6_ − 4.53 A_665.6_; total carotenoids = 1000 A_480_ − 1.33Ca − 23.93Cb)/202, according to the method standardized by Wellburn [47].

#### 4.4.3. Chromatographic Analysis of the Phenolic Composition

The phenolic composition of the tested extracts was evaluated by both HPTLC and HPLC analysis, as previously reported [46,48]. In particular, HPTLC analysis was carried out by a CAMAG HPTLC system (Muttenz, Switzerland), controlled by the WinCATS 1.4.4 Planar Chromatography Manager (CAMAG) software. Phenolics in the extracts were identified by the comparison of different parameters (Rf values, colors, UV spectra) of the selected standards (chlorogenic acid, caffeic acid, gallic acid, rutin, quercetin, kaempferol, apigenin, luteolin, and catechin) and data from the literature. HPLC-PDA analysis was performed according to previous standardized methods [46]. To perform the analysis, a Waters HPLC liquid chromatography (model 600 solvent pump, 2996 PDA) connected to a C18 reversed-phase column (Prodigy ODS-3, 4.6 × 150 mm, 5 μm; Phenomenex, Torrance, CA, USA), was used. A panel of fifteen pure phenolic compounds, including benzoic acid, caffeic acid, chlorogenic acid, cinnamic acid, coumaric acid, ferulic acid, gallic acid, syringic acid, catechin, epicatechin, hesperetin, 3-hydroxyflavone, quercetin, rutin, and syringaldehyde was determined in the extracts. To assure repeatability, the analyses were repeated at least twice.

#### 4.4.4. Gas Chromatography–Mass Spectrometry (GC–MS) Analysis

The untargeted metabolomic analysis of tested extracts was carried out by GC–MS after TBDMS derivatization, according to previous methods [49]. Briefly, 1 mg of the dry sample was dissolved in pyridine (50 µL), and neat MTBSTFA (50 μL) was added. The mixture was heated at 80 °C for 1 h. The sample was dried under a stream of N_2_, and the residue was resuspended in 0.1 mL of dichloromethane for GC–MS analysis. GC–MS analyses were performed using an Agilent 7890B gas chromatograph coupled to a 5977B quadrupole mass selective detector (Agilent Technologies, Palo Alto, CA, USA). Chromatographic separations were carried out with an Agilent HP5 ms fused-silica capillary column (30 m × 0.25 mm i.d.) coated with 5%-phenyl-95%-dimethylpolysiloxane (film thickness 0.25 μm) as the stationary phase. Injection mode: splitless at a temperature of 280 °C. Column temperature program: 70 °C (1 min) then to 300 °C at a rate of 20 °C/min and held for 10 min. The carrier gas was helium at a constant flow of 1.0 mL/min. The spectra were obtained in the electron impact mode at 70 eV ionization energy; ion source 280 °C; ion source vacuum 10–5 Torr. MS analysis was performed in TIC (mass range scan from *m*/*z* 50 to 600 at a rate of 0.42 scans s-1). Identification was made by comparison with the fragmentation profiles from the NIST2017 database.

### 4.5. Radical Scavenging Activity

DPPH- and ABTS-radical scavenging activities were determined according to the previously described spectrophotometric methods [50], with minor changes. For both assays, the percentage of scavenger activity was calculated as follows: 100 × (A_control_ − A_sample_)/A_control_, where A_control_ is the absorbance of the radical alone, while A_sample_ is that of radical with sample.

### 4.6. Iron Chelating Activity

The chelating abilities of the tested extracts were evaluated by the ferrozine assay against ferrous ions according to previous published methods [46]. The absorbance of the ferrous ions and ferrozine complex was measured spectrophotometrically at 562 nm. The percentage of chelating activity was calculated as follows: 100 (A_control_ − A_sample_)/A_control_, where A_control_ is the absorbance of the vehicle whereas A_sample_ is that of the tested sample. For each sample, the iron-chelating power was calculated in relation with the positive control rutin.

### 4.7. Inhibition of Advanced Glycation End-Product (AGE)

The ability of the tested samples (1–1000 μg/mL) to block the AGE formation was measured according to the fluorescence method reported by Di Sotto et al. and compared with the standard antiglycation agent rutin [51]. Fluorescence was measured at an excitation wavelength of 355 nm and an emission of 460 nm. The inhibitory activity was calculated as percentage of the control by using the following formula: (A_control_ − A_sample_/A_control_) 100, where A_control_ is the fluorescence of the control, whereas A_sample_ is the fluorescence of the sample.

### 4.8. In Vitro Metabolic Enzyme Inhibition

The ability of the extracts to inhibit the α-amylase and α-glucosidase enzymes was measured spectrophotometrically by using a microplate reader (Epoch Microplate Spectrophotometer, BioTeK, Winooski, VT, USA) [46]. Acarbose was used as a standard inhibitor (100% enzyme inhibition), while the vehicle was considered as the maximum enzyme activity. Additional treatments, wherein buffer solution was added instead of enzyme, were included in order to measure any possible interfering absorbance of the samples. At least three repeated experiments and six replicates for each experiment were performed and data obtained were pooled for the statistical analysis. The inhibitory activity was calculated as the percentage of inhibition with respect to the control.

#### 4.8.1. α-Amylase Inhibition

The α-amylase activity was determined by the method of dinitrosalicilic acid (DNSA). To perform the assay, serial dilutions of the extract (250 μL; 1–1000 μg/mL) were pre-incubated with the enzyme (250 μL; 0.5 mg/mL in phosphate buffer solution, corresponding to 25 U/mL) for 10 min at 37 °C. Next, a potato starch solution (0.5% *w*/*v* in acetate buffer 0.1 M, pH 4.5; 250 μL) was added to the mixture, and the mixture was further incubated for 10 min at 37 °C. Thereafter, adding 500 μL of DNSA reagent (25 mL of 96 mM DNSA solution in water, 8 mL of 5.3 M sodium potassium tartrate solution in 2 M sodium hydroxide and 12 mL of water) and further incubating the samples in a water bath at 100 °C for 5 min led to the development of a red color (due to the binding of DNSA and reducing sugar in the mixture), which was measured at 540 nm.

#### 4.8.2. α-Glucosidase Inhibition

The method based on the enzymatic hydrolysis of p-nitrophenyl-α-D-glucopiranoside (PNGP) to p-nitrophenol and D-glucose was applied [49]. To perform the assay, serial dilutions of the extracts (25 μL; 1–1000 μg/mL) were pre-incubated for 30 min at 37 °C with enzyme (50 μL; 1 U/mL in PBS 0.1 M) and 100 μL of PBS. After mixing with PNGP (25 μL; 5 mM in PBS) for 5 min, p-nitrophenol was formed and its absorbance was measured at 405 nm.

### 4.9. Antimicrobial Activity

#### 4.9.1. Microorganisms

Eight bacterial, four yeast, and eight dermatophyte strains were obtained from the Mycology Laboratory (Department of Chemistry, Biology and Biotechnology, Perugia University, Italy). The reference strains of microorganisms from the American Type Culture Collection (ATCC), Manassas (USA); Culture Colletion of Fungi (CCF), Charles University, Prague (Czech Republic); and Industrial Yeasts Collection DBVPG, Perugia University (Italy), were included. The bacterial strains include *Escherichia coli* ATTC 10536, *E. coli* 1 (environmental isolate), *E. coli* 2 (environmental isolate), *Bacillis cereus* ATCC 12826, *Pseudomonas aeruginosa* ATTC 15442, *Bacillus subtilis* (clinical isolate), *Salmonella typhi* (clinical isolate), and *Staphylococcus aureus* ATCC 6538. The yeast and dermatophyte strains were *Candida tropicalis* YEPGA 6184, *C. albicans* YEPGA 6379, *C. parapsilosis* YEPGA 6551, *C. albicans* YEPGA 6183, *T. tonsurans* CCF 4834, *T. rubrum* CCF 4933, *T. erinacei* CCF 6261, *Arthroderma quadrifidum* CCF 5792, *A. curreyi* CCF 5207, *A. interdifitale* CCF 823, and *Nannizia gypsea* CCF 6261.

#### 4.9.2. Antibacterial Activity Assay

The minimal inhibitory concentration (MIC) values were determined as described in the Clinical and Laboratory Standards Institute (CLSI) guidelines [52]. The determination was performed in 96-well-microtiter plates (Sarsted, Milan, Italy) using concentrations of the extract in the range of 50–6.25 µg/mL, derived from serial two-fold dilutions in Mueller–Hinton Broth (MHB) with a bacterial inoculum of about 106 CFU/mL. The set-up included bacterial growth controls in wells containing 10 μL of the test inoculum and negative controls without bacterial inoculum. The MIC end-points were determined after 18–20 h of incubation in ambient air at 35 °C [52]. For plant extract and ciprofloxacin, the MIC end-points were defined as the lowest concentration that totally inhibited bacterial growth [53]. Each test was done in triplicate. Geometric means and MIC ranges were calculated.

#### 4.9.3. Antifungal Activity Assay

In vitro susceptibility testing against yeasts and dermatophytes was performed by a broth microdilution technique using 96-well-microplates (Sarsted, Milan, Italy) according to M27-A3, M38-A2, and supplement M61 documents published by the Clinical and Laboratory Standards Institute [54,55,56,57]. *Candida parapsilosis* ATCC 22019 and *Candida krusei* ATCC 6258 strains were used as quality controls [54,55,56,57]. For the Minimum Inhibitory Concentration (MIC) determination, the plant extract had a MIC in a range of 1.56–200 μg/mL. RPMI (Roswell Park Memorial Institute) 1640 medium supplemented with 2% glucose (w/v) and buffered with 0.165 M MOPS [3-( N-morpholine) propanesulphonic acid], containing L-glutamine and without sodium bicarbonate, was dissolved in 1 L of sterile distilled water, adjusted to pH 7 and filter sterilized. All microbial cultures used were first subcultured on Sabouraud agar (Sigma-Aldrich, Milan, Italy). Inoculum suspensions were prepared in a sterile saline solution (0.85% NaCl) with an optical density of 0.5 Mac Farland standard. In brief, 10 µL of the suspension was inoculated into each well of a 96-well microplate containing 150 µL of RPMI medium with a twofold-diluted concentration of the extract [58]. MIC end-points (µg/mL) were determined after 24 h (for yeasts) and 48–72 h (for dermatophytes) of incubation in ambient air at 30 °C [56,57]. For extract and fluconazole, the MIC end-point was defined as the lowest concentration that prevented any visually discernible growth. Each test was done in triplicate. Geometric means and MIC ranges were calculated [53].

### 4.10. Bioinformatics

Putative targets of antimicrobial activity were identified according to the bioinformatics method described by Gu and colleagues [59]. Briefly, proteins targeted by extracts were predicted using the bioinformatics platform STITCH; the same resource was employed for the network-pharmacology and KEGG analyses. Docking runs were conducted as previously described [60]. Crystal structures of target proteins were derived from the Protein Data Bank (PDB) with PDB ID as follows: 1B2Y (α-amylase) and 3WY1 (α-glucosidase). Discovery studio 2020 visualizer was employed to investigate the protein–ligand nonbonding interactions. Finally, the putative toxicity of extracts’ phytochemicals was predicted in the *Daphnia magna* model through the Toxicity Estimation Software Tool (T.E.S.T.), and the results were expressed as LC_50_ values, whereas the pharmacokinetics was predicted using the SwissADME platform.

### 4.11. Statistical Analysis

Data are reported as means ± SEM. Statistical analysis was performed by GraphPad Prism™ (Version 6.00) software (GraphPad Software, Inc., San Diego, CA, USA). A significant difference among treatments was established by the one-way analysis of variance (one-way ANOVA), followed by Dunnett’s Multiple Comparison Post Test. The concentration–response curves were obtained by nonlinear regression through the “Hill equation”: E = Emax/[1 + (10LogIC_50_/A) ^HillSlope^], where E is the effect at a given concentration of agonist, Emax is the maximum activity, IC_50_ is the concentration that produces 50% of the inhibitory response, A is the agonist concentration in molarity, and HillSlope is the slope of the agonist curve. Less than 0.05 (*p* < 0.05) *p* values were considered as statistically significant.

## 5. Conclusions

The present study provides preliminary scientific evidence about the hypoglycemic, antiglycative, and antibacterial properties of the extracts from the Moroccan *Anacyclus maroccanus* Ball and *Anacyclus radiatus* Loisel ecotypes and suggests the need for further studies to clarify the mechanisms involved and the in vivo efficacy. To the best of our knowledge, this is the first report highlighting the phytochemical composition and the potential biological properties of these species; indeed, *A. maroccanus* had not been studied until now. Moreover, the evidence about the bacteriostatic and mycostatic effects of the AR extracts supports, at least in part, the ethnopharmacological use of *A. radiatus* aerial parts as remedies for microbial infections. Altogether, the present results suggest a further interest in these species as natural sources of bioactive compounds and/or phytocomplexes, which could enter pharmaceutical and nutraceutical process development, with possible benefits for Moroccan economy.

## Figures and Tables

**Table 1:** Total amount of polyphenols, tannins, and flavonoids from *Anacyclus maroccanus* Ball (AM) and *Anacyclus radiatus* Loisel (AR) dry extracts, determined as tannic acid equivalents (TAE) and quercetin equivalents (QE). Data are expressed as means ± standard error (SE) of at least two experiments and six replicates (*n* = 6).

Samples	Polyphenols	Tannins	Flavonoids
	mg TAE/g Dry Extract		mg QE/g Dry Extract
AM extracts			
Methanolic	138.0 ± 6.1 ***	36.7 ± 2.9 ***	62.9 ± 1.2 ***
Ethyl acetate	67.7 ± 4.2	14.6 ± 2.7	21.0 ± 1.4
AR extracts			
Methanolic	220.3 ± 6.7 ***°°°	53.2 ± 6.9 ***°°	30.2 ± 1.1 **°°°
Ethyl acetate	76.2 ± 4.6 °	28.7 ± 2.2 °°°	20.6 ± 1.0

** *p* < 0.01 and *** *p* < 0.001 denote a significant difference in the amounts determined in the methanolic extract with respect to the ethyl acetate extract (*t*-Student Test); ° *p* < 0.05, °° *p* < 0.01, and °°° *p* < 0.001 denote a significant difference in the amounts determined in the AR extracts with respect to the AM extracts (*t*-Student Test).

**Table 2:** Total chlorophyll A, B, and carotenoid content, as well as pigment ratios, of *Anacyclus maroccanus* Ball (AM) and *Anacyclus radiatus* Loisel (AR) dry extracts. Values are expressed as means ± standard error (*n* = 6).

Samples	Carotenoids	Chlorophyll A (Chl A)	Chlorophyll B (Chl B)	Pigment Ratios		
	µg/g of Dry Extract			Chl A/Chl B	Chl A + Chl B	Chlorophylls/Carotenoids
AM extracts						
Methanolic	172.53 ± 1.33	62.92 ± 0.28	149.81 ± 0.47	0.42	2.66	1.22
Ethyl acetate	89.52 ± 1.75 ***	283.80 ± 3.47 ***	606.77 ± 5.04 ***	0.47	11.13	9.95
AR extracts						
Methanolic	434.62 ± 3.34 °°°	59.56 ± 1.16	198.83 ± 0.82 °°	0.31 °	3.23 °	0.59 °°°
Ethyl acetate	129.33 ± 0.46 ***°°°	105.23 ± 2.51 ***°°°	333.96 ± 1.30 ***°°°	0.32 °	5.49 °°°	3.39 °°°

*** *p* < 0.001 denotes a significant difference in the amounts determined in the methanolic extract with respect to the ethyl acetate extract (*t*-Student Test); ° *p* < 0.05, °° *p* < 0.01, and °°° *p* < 0.001 denote a significant difference in the amounts determined in the AR extracts with respect to the AM extracts (*t*-Student Test).

**Table 3:** HPLC analysis of *Anacyclus maroccanus* Ball (AM) and *Anacyclus radiatus* Loisel (AR) dry extracts. Values are expressed as means ± standard error (*n* = 6).

Compound	AM Extracts		AR Extracts	
	Methanolic	Ethyl Acetate	Methanolic	Ethyl Acetate
	mg/g of Dry Extract			
3-Hydroxyflavone	-	-	-	-
Benzoic Acid	0.1 ± 0.01	2.03 ± 0.02 ***	4.86 ± 0.04 °°°	20.60 ± 0.47 ***°°°
Caffeic Acid	0.26 ± 0.01	1.49 ± 0.01 ***	0.61 ± 0.01 °°°	2.22 ± 0.22 ***
Catechin	0.76 ± 0.01	0.71 ± 0.03 ***	0.49 ± 0.01	3.44 ± 0.02 ***
Chlorogenic Acid	10.38 ± 0.21	8.68 ± 0.01 ***	16.82 ± 0.11 °°°	11.55 ± 0.02 °°°
Cinnamic Acid	-	-	-	-
Coumaric Acid	0.03 ± 0.02	-	0.12 ± 0.01 °°°	0. 26 ± 0.02 ***°°°
Epicatechin	4.64 ± 0.01	7.64 ± 0.06 ***	4.90 ± 0.01	16.55 ± 0.31 ***°°°
Ferulic Acid	0.03 ± 0.01	0.49 ± 0.01 ***	0.74 ± 0.03 °°°	2.64 ± 0.02 ***°°°
Gallic Acid	0.1 ± 0.01	0.37 ± 0.01 ***	0.29 ± 0.01 °°°	0.76 ± 0.01 ***°°°
Hesperidin	0.03 ± 0.01	0.34 ± 0.01 ***	-	-
Quercetin	0.1 ± 0.01	1.40 ± 0.01 ***	0.26 ± 0.01 °°°	1.54 ± 0.26 ***
Rutin	2.37 ± 0.01	27.23 ± 0.35 ***	1.23 ± 0.01 °°°	33.91 ± 0.55 ***°
Syringaldehyde	-	1.92 ± 0.01 ***	1.25 ± 0.01 °°°	3.55 ± 0.18 ***°°°
Syringic Acid	0.27 ± 0.01	0.66 ± 0.01 ***	0.56 ± 0.01 °°°	1. 16 ± 0.05 ***°°°

*** *p* < 0.001 denotes a significant difference in the amounts determined in the methanolic extract with respect to the ethyl acetate extract (*t*-Student Test); ° *p* > 0.05 and °°° *p* < 0.001 denote a significant difference in the amounts determined in the AR extracts with respect to AM extracts (*t*-Student Test).

**Table 4:** GC-MS analysis of *Anacyclus maroccanus* Ball (AM) and *Anacyclus radiatus* Loisel (AR) dry extracts. Values are expressed as means ± standard error (*n* = 6).

Compounds	Retention Time	AM Extracts		AR Extracts	
		Methanolic	Ethyl Acetate	Methanolic	Ethyl Acetate
		Area %			
Dicarboxylic Acids					
Azelaic acid	19.28	2.764	5.520	2.540	3.689
Butanedioic acid	14.36	3.518	9.982	3.686	7.438
Meglutol	22.50	29.255	16.499	25.425	5.790
Methylmalonic acid	14.50	5.108	2.388	8.936	2.819
Sebacic acid	20.13	0.617	0.461	0.603	0.466
Suberic acid	18.36	0.238	0.845	0.255	0.658
Alpha-Hydroxy Acids					
Citric acid	22.10	2.894	0.468	1.570	0.803
Glycolic acid	11.45	0.561	0.421	0.962	0.597
Lactic acid	11.24	0.444	0.639	0.947	1.052
Hydroxyisocaproic acid	15.48	0.813	1.086	0.710	1.269
Phenolic Acids					
Hydroxycinnamic acids					
Caffeic acid	23.82	2.337	13.58	5.521	22.218
Coumaric acid	20.83	2.267	5.189	2.259	6.028
Ferulic acid	22.08	15.095	3.662	1.419	0.919
Hydroxybenzoic acids					
Benzenacetic acid	11.69	0.019	0.294	0.067	0.44
Benzoic acid	11.23	0.111	0.354	0.477	0.151
2,5-Dihydroxybenzoic acid	20.71	1.606	0.652	1.817	1.015
Gallic acid	23.53	0.431	0.447	0.751	2.138
Ketoglutaric acid	14.61	0.146	0.455	1.437	0.373
Isovanillic acid	19.14	1.876	2.099	1.930	6.820
Methoxysalicylic acid	18.67	0.968	0.858	0.797	1.038
Protocatechuic acid	21.41	1.161	4.403	1.389	6.462
Salicylic acid	16.50	1.263	2.246	0.511	1.782
Fatty acids					
Behenic acid	23.98	0.448	0.600	1.004	0.663
Levulinic acid	9.59	0.164	0.895	0.166	0.507
α-Linoleic acid	20.89	1.863	2.339	0.573	1.983
Myristic acid	16.18	4.692	1.121	7.143	0.857
Octenoic acid	11.80	0.133	0.790	0.406	0.585
Octadecadienoic acid	15.77	0.118	0.747	0.146	0.359
Palmitic acid	19.40	1.232	6.718	1.767	6.049
Pentanedioic acid	15.36	0.180	0.705	0.253	1.107
Pentenedioic acid	15.95	0.352	2.647	0.239	2.148
Propanedioic acid	13.07	0.419	0.938	2.133	0.785
Propanoic acid	12.54	0.175	0.735	0.667	0.933
Stearic acid	21.04	1.210	0.810	1.222	0.697
Tetradecanedioic acid	20.34	0.865	0.889	1.776	3.642
Miscellaneous					
Glycerol	15.63	4.951	1.436	8.354	2.185
Isonicotinic acid	11.84	1.384	0.420	2.098	0.508
Phosphoric acid	16.07	1.314	0.710	1.019	0.854
L-Pyroglutamic acid	16.36	6.330	2.353	6.014	1.021
Urea	13.17	0.554	2.253	0.722	0.991
Vanillic aldehyde	14.70	0.123	0.347	0.288	0.159

**Table 5:** IC_50_ values (µg/mL) of *Anacyclus maroccanus* Ball (AM) and *Anacyclus radiatus* Loisel (AR) dry extracts and the positive control in the radical scavenging assays. Values are expressed as means ± standard error (*n* = 6).

Samples	Radical Scavenging Activity	
	DPPH	ABTS
	IC_50_ (CL ^a^) μg/mL	
AM extracts		
Methanolic	126.9 (90.3–178.4)	29.3 (17.7–48.6)
Ethyl acetate	18.6 (17.6–19.7) ***	6.3 (3.1–13.1) ***
AR extracts		
Methanolic	19.3 (5.4–69.8) °°°***	19.0 (4.9–73.5)
Ethyl acetate	5.3 (2.2–13.0) °°°***	8.0 (2.1–28.5) **°
Positive control ^b^	8.9 (1.5–24.8)	1.8 (1.4–2.3)

^a^ CL, confidence limit. ^b^ Trolox. ** *p* > 0.01 and *** *p* < 0.001 denote a significant difference between the ethyl acetate and methanolic extracts in each species (*t*-Student Test); ° *p* > 0.05 and °°° *p* < 0.001 denote a significant difference compared to the respective AM extract (*t*-Student Test).

**Table 6:** IC_50_ values (µg/mL) of *Anacyclus maroccanus* Ball (AM) and *Anacyclus radiatus* Loisel (AR) dry extracts and the positive control rutin in advanced glycation end-products (AGE) inhibition and in ferrous ion chelating activity assays. Values are expressed as means ± standard error (*n* = 6).

Samples	Inhibition of Advanced Glycation End-Products (AGE)	Ferrous ion Chelating Activity
	IC_50_ (CL ^a^) μg/mL	
AM extracts		
Methanolic	ne ^b^	265.4 (119.4–590.2)
Ethyl acetate	73.3 (39.7–135.3)	153.3 (105.4–223.0)
AR extracts		
Methanolic	ne ^b^	602.1 (407.4–889.7)
Ethyl acetate	41.2 (24.0–70.6)	268.4 (159.6–451.2)
Positive control ^c^	2.8 (2.2–3.5)	555.8 (386.6–799.0)

^a^ CL, confidence limit; ^b^ ne, not evaluable being the achieved effect lower than 80%; ^c^ Rutin.

**Table 7:** IC_50_ values (µg/mL) of the dry extracts from *Anacyclus maroccanus* and *Anacyclus radiatus* and the positive control acarbose for α-amylase and α-glucosidase enzyme inhibition.

Samples	α-Amylase Inhibition	α-Glucosidase Inhibition
	IC_50_ (CL ^a^) μg/mL	
AM extracts		
Methanolic	49.5 (23.3–147.1)	ne ^b^
Ethyl acetate	15.1 (4.6–98.4)	246.6 (95.9–789.0)
AR extracts		
Methanolic	ne ^b^	ne ^b^
Ethyl acetate	ne ^b^	143.8 (73.8–527.0)
Positive control ^c^	82.2 (15.0–450.6)	1.3 (0.9–5.5)

^a^ CL: confidence limit; ^b^ ne, not evaluable being the maximum achieved effect lower than 80%; ^c^ Acarbose.

**Table 8:** Minimal inhibitory concentrations (MICs) of the methanolic and ethyl acetate dry extracts from *Anacyclus maroccanus* Ball (AM) and *Anacyclus radiatus* Loisel (AR) against bacteria isolates.

	AM Extract		AR Extract	
	Methanolic	Ethyl Acetate	Methanolic	Ethyl Acetate
Bacteria	MIC (µg/mL) *			
*Escherichia coli* (ATCC 10536)	31.94 (25–50)	19.84 (12.5–25)	15.75 (12.5–25)	9.92 (6.25–12.5)
*Escherichia coli* (environm. isolate 1)	9.92 (6.25–12.5)	7.87 (6.25–12.5)	<6.25	7.87 (6.25–12.5)
*Escherichia coli* (environm. isolate 2)	19.84 (12.5–25)	31.49 (25–50)	15.75 (12.5–25)	19.84 (12.5–25)
*Bacillus cereus* (ATCC 12826)	31.49 (25–50)	39.68 (25–50)	19.84 (12.5–25)	31.49 (25–50)
*Pseudomonas aeruginosa* (ATCC 15442)	31.49 (25–50)	31.49 (25–50)	15.75 (12.5–25)	19.84 (12.5–25)
*Bacillus subtilis* (clinical isolate)	19.84 (12.5–25)	15.75 (12.5–25)	9.92 (6.25–12.5)	9.92 (6.25–12.5)
*Salmonella typhi* (clinical isolate)	31.49 (25–50)	39.68 (25–50)	19.84 (12.5–25)	15.75 (12.5–25)
*Staphylococcus aureus* (ATCC 6538)	19.84 (12.5–25)	15.75 (12.5–25)	9.92 (6.25–12.5)	15.75 (12.5–25)

* Mic values are reported as geometric means of three independent replicates (*n* = 3). MIC range concentrations are reported within brackets.

**Table 9:** Minimal inhibitory concentrations (MICs) of the methanolic and ethyl acetate dry extracts from *Anacyclus maroccanus* Ball (AM) and *Anacyclus radiatus* Loisel (AR) against yeast isolates.

	AM Extract		AR Extract	
	Methanolic	Ethyl Acetate	Methanolic	Ethyl Acetate
Yeast Strain	MIC (µg/mL) *			
*Candida tropicalis* (YEPGA 6184)	125.99 (100–200)	125.99 (100–200)	125.99 (100–200)	158.74 (100–200)
*Candida albicans* (YEPGA 6379)	125.99 (100–200)	125.99 (100–200)	79.37 (50–100)	125.99 (100–200)
*Candida parapsilosis* (YEPGA 6551)	158.74 (100–200)	79.37 (50–100)	62.99 (50–100)	79.37 (50–100)
*Candida albicans* (YEPGA 6183)	79.37 (50–100)	62.99 (50–100)	39.68 (25–50)	79.37 (50–100)

* Mic values are reported as geometric means of three independent replicates (*n* = 3). MIC range concentrations are reported within brackets.

**Table 10:** Minimal inhibitory concentrations (MICs) of the methanolic and ethyl acetate dry extracts from *Anacyclus maroccanus* Ball (AM) and *Anacyclus radiatus* Loisel (AR) against dermatophyte isolates.

	AM Extract		AR Extract	
	Methanolic	Ethyl Acetate	Methanolic	Ethyl Acetate
Dermatophytes	MIC (µg/mL) *			
*Trichophyton interdigitale* (CCF 4823)	125.99 (100–200)	79.37 (50–100)	79.37 (50–100)	31.49 (25–50)
*Trichophyton tonsurans* (CCF 4834)	31.49 (25–50)	39.68 (25–50)	9.92 (6.25–12.5)	9.92 (6.25–12.5)
*Trichophyton rubrum* (CCF 4933)	39.68 (25–50)	19.84 (12.5–25)	19.84 (12.5–25)	9.92 (6.25–12.5)
*Arthroderma quadrifidum* (CCF 5792)	79.37 (50–100)	158.74 (100–200)	39.68 (25–50)	79.37 (50–100)
*Trichophyton erinacei* (CCF 5930)	125.99 (100–200)	>200	>200	62.99 (50–100)
*Nannizia gypsea* (CCF 6261)	31.49 (25–50)	19.84 (12.5–25)	39.68 (25–50)	19.84 (12.5–25)
*Arthroderma curreyi* (CCF 5207)	39.68 (25–50)	9.92 (6.25–12.5)	31.49 (25–50)	19.84 (12.5–25)

* Mic values are reported as geometric means of three independent replicates (*n* = 3). MIC range concentrations are reported within brackets.

## Data Availability

Data available within the article and its supplementary materials.

## Supplementary Materials

The following supporting information can be downloaded online. Figure S1: High-performance thin-layer chromatography (HPTLC) analysis of *Anacyclus maroccanus* Ball (AM) and *Anacyclus radiatus* Loisel (AR) extracts; Figure S2: HPLC-DAD chromatograms of *Anacyclus maroccanus* Ball (AM) and *Anacyclus radiatus* Loisel (AR) extracts; Figure S3. GC-MS chromatograms of *Anacyclus maroccanus* Ball (AM) and *Anacyclus radiatus* Loisel (AR) extracts; Figure S4: (A) Putative interactions between rutin and α-amylase (PDB: 1B2Y). Free energy of binding (ΔG) and affinity (Ki) are −9.3 kcal/mol and 180 nM, respectively. (B) Putative interactions between rutin and α-glucosidase (PDB: 1B2Y).

## Sample Availability

Samples of the tested AM and AR extracts are available from the authors.
