# Supplementary material for: Characterization of the Phytochemical Composition and Bioactivities of Anacyclus maroccanus Ball. and Anacyclus radiatus Loisel Aerial Parts: Preliminary Evidence for the Possible Development of Moroccan Plants
Source: Molecules. 2022 Jan 21;27(3):692. doi: 10.3390/molecules27030692 (PMC8839974; doi:10.3390/molecules27030692)
Supplement: Supplementary file 1 [file molecules-27-00692-s001.zip › molecules-1530541-supplementary.pdf]

molecules

Supplementary materials 1

Characterization of the Phytochemical Composition and Bioactivities of *Anacyclus maroccanus* Ball. and *Anacyclus radiatus* Loisel Aerial Parts: Preliminary Evidence for the Possible Development of Moroccan Plants

Saida Sissi [1,2], Silvia Di Giacomo [2], Claudio Ferrante [3], Paola Angelini [4], Alberto Macone [5], Anna Maria Giusti [6], Chiara Toniolo [7], Annabella Vitalone [2], Aghraz Abdellah [1], Mustapha Larhsini [1], Luigi Menghini [3], Mohammed Markouk [1], Gabriela Mazzanti [2,*] and Antonella Di Sotto [2,*]

1 Laboratory of Agri-Food, Biotechnology, and Valorization of Plant Resources, Phytochemistry and Pharmacology of Medicinal Plants Unit, Faculty of Sciences Semlalia, Cadi Ayyad University, P.O. Box 2390, Marrakech 40000, Morocco; saida.sissi@ced.uca.ma (S.S.); abdellah.aghraz@gmail.com (A.A.); larhsini@uca.ac.ma (M.L.); markouk@uca.ac.ma (M.M.)
2 Department of Physiology and Pharmacology "V. Erspamer", Sapienza University of Rome, P.le Aldo Moro 5, 00185 Rome, Italy; silvia.digiacomo@uniroma1.it (S.D.G.); annabella.vitalone@uniroma1.it (A.V.)
3 Department of Pharmacy, Botanic Garden "Giardino dei Semplici", University "Gabriele d'Annunzio" of Chieti-Pescara, Via dei Vestini 31, 66100 Chieti, Italy; claudio.ferrante@unich.it (C.F.); luigi.menghini@unich.it (L.M.)
4 Department of Chemistry, Biology and Biotechnology, University of Perugia, 06122 Perugia, Italy; paola.angelini@unipg.it
5 Department of Biochemical Sciences, A. Rossi Fanelli, Sapienza University of Rome, P.le A. Moro 5, 00185 Rome, Italy; alberto.macone@uniroma1.it
6 Department of Experimental Medicine, Sapienza University of Rome, P.le Aldo Moro 5, 00185 Rome, Italy; annamaria.giusti@uniroma1.it
7 Department of Environmental Biology, Sapienza University of Rome, P.le Aldo Moro 5, 00185 Rome, Italy; chiara.toniolo@uniroma1.it
* Correspondence: gabriela.mazzanti@uniroma1.it (G.M.); antonella.disotto@uniroma1.it (A.D.S.)

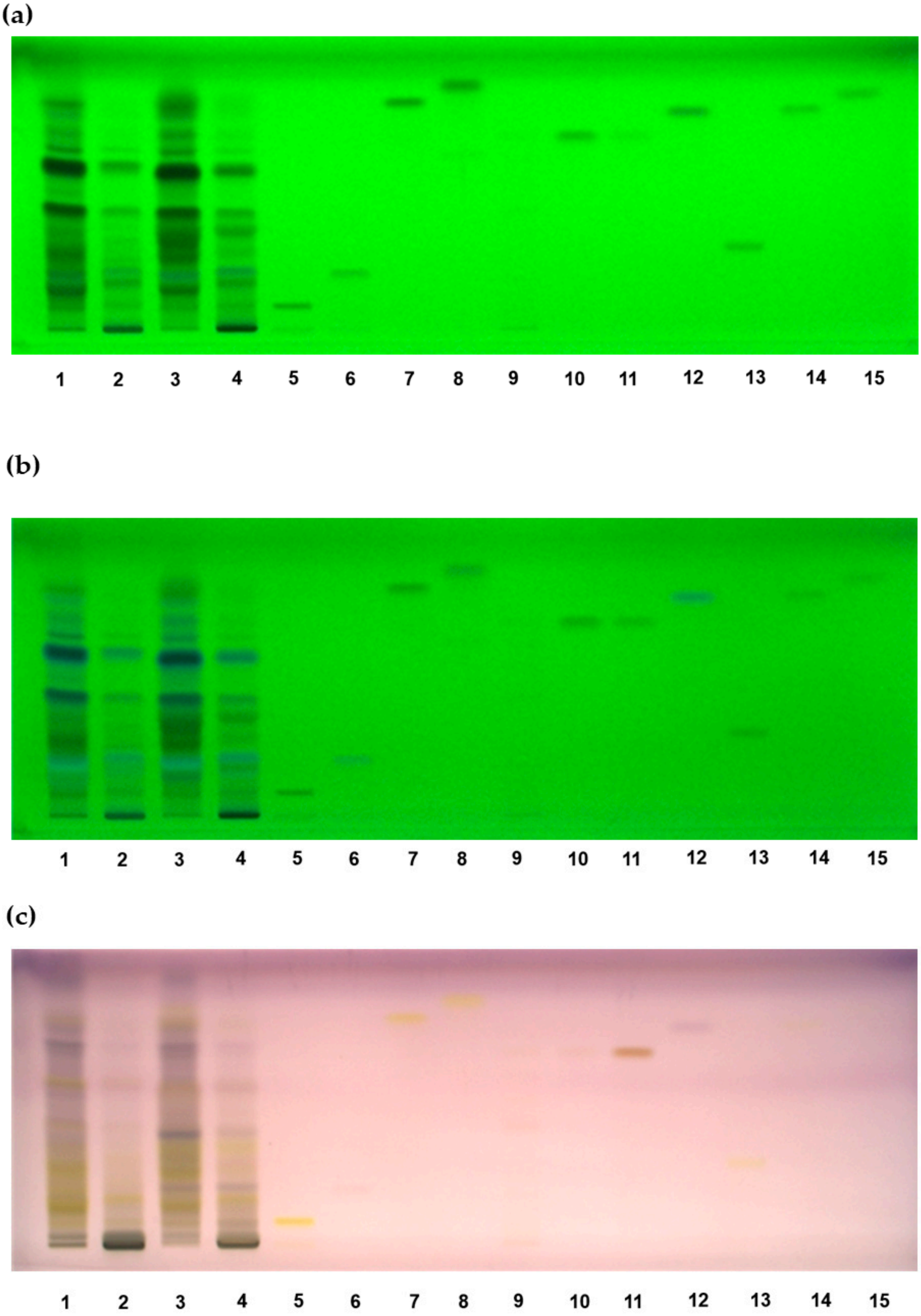
(a)
1 2 3 4 5 6 7 8 9 10 11 12 13 14 15
(b)
1 2 3 4 5 6 7 8 9 10 11 12 13 14 15
(c)
1 2 3 4 5 6 7 8 9 10 11 12 13 14 15

(d)

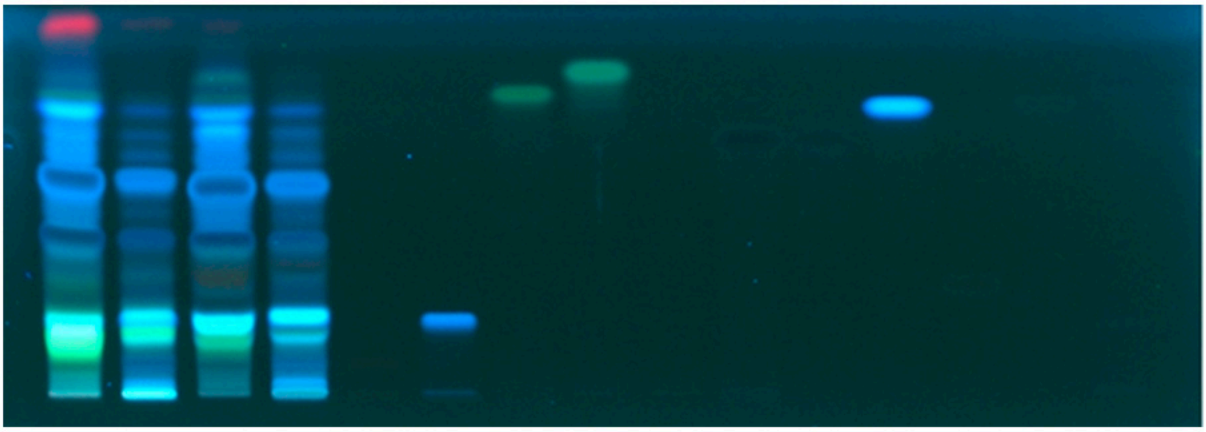

(e)

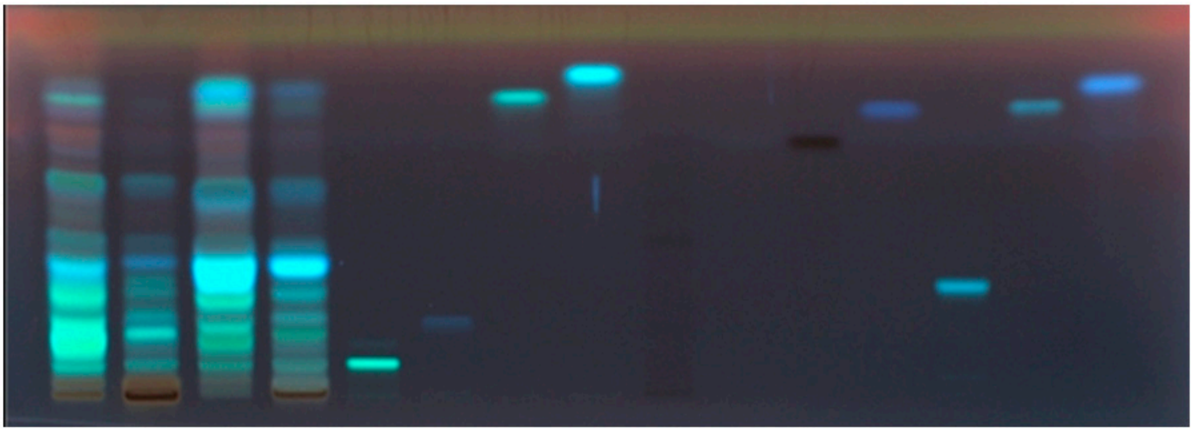

Figure S1. High-performance thin-layer chromatography (HPTLC) analysis of Anacyclus maroccanus Ball (AM) and Anacyclus radiatus Loisel (AR) extracts. HPTLC silica gel 60 glass-backed layers were developed using the solvent mixture ethyl acetate:dichloromethane:acetic acid:formic acid:water (100:25:10:10:11 v/v/v/v/v) and derivatized by Natural Product Reagent (NPR) and/or anisaldehyde. (a) Visualization at 254 nm. (b) Visualization at 254 nm after Natural Product Reagent (NPR) derivatization. (c) Visualization under white light after NPR and anisaldehyde derivatization (d) Visualization at 366 nm without derivatization. (e) Visualization at 366 nm after Natural Product Reagent (NPR) derivatization. The chromatograms correspond to 1. AM ethyl acetate extract; 2. AM methanolic extract; 3. AR ethyl acetate extract; 4. AR methanolic extract; 5. rutin; 6. chlorogenic acid; 7. quercetin; 8. kampferol; 9. epigallocatehin; 10. gallic acid; 11 catechin; 12. caffeic acid; 13. luteolin 7-O-glucoside; 14; luteolin; 15. apigenin.

(a)

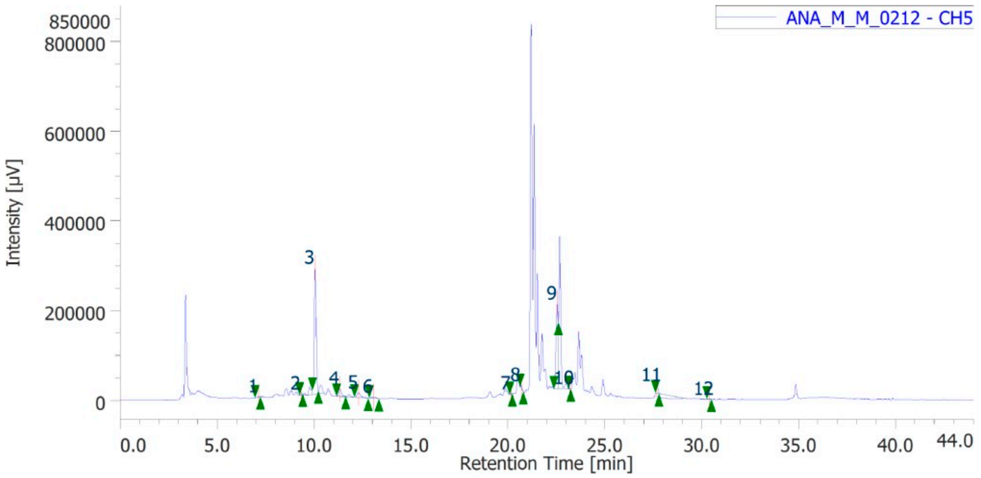
ANA_M_M_0212 - CH5
Intensity [µV]
850000
800000
600000
400000
200000
0
Retention Time [min]
0.0
5.0
10.0
15.0
20.0
25.0
30.0
35.0
40.0
44.0

(b)

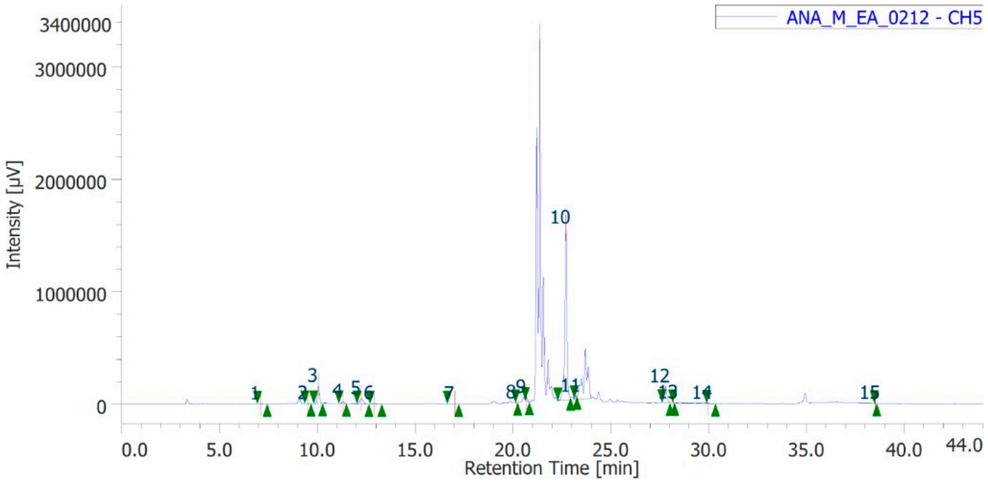
ANA_M_EA_0212 - CH5
Intensity [µV]
3400000
3000000
2000000
1000000
0
Retention Time [min]
0.0
5.0
10.0
15.0
20.0
25.0
30.0
35.0
40.0
44.0

(c)

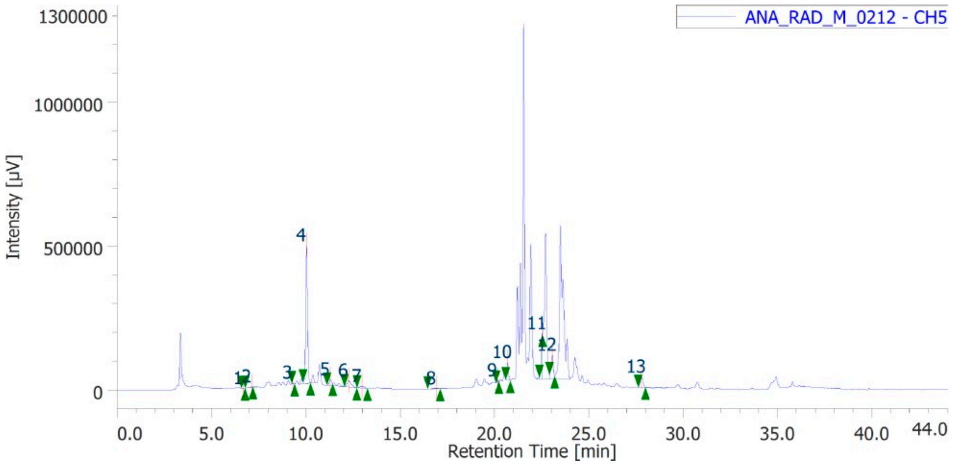
ANA_RAD_M_0212 - CH5
Intensity [µV]
1300000
1000000
500000
0
Retention Time [min]
0.0
5.0
10.0
15.0
20.0
25.0
30.0
35.0
40.0
44.0

(d)

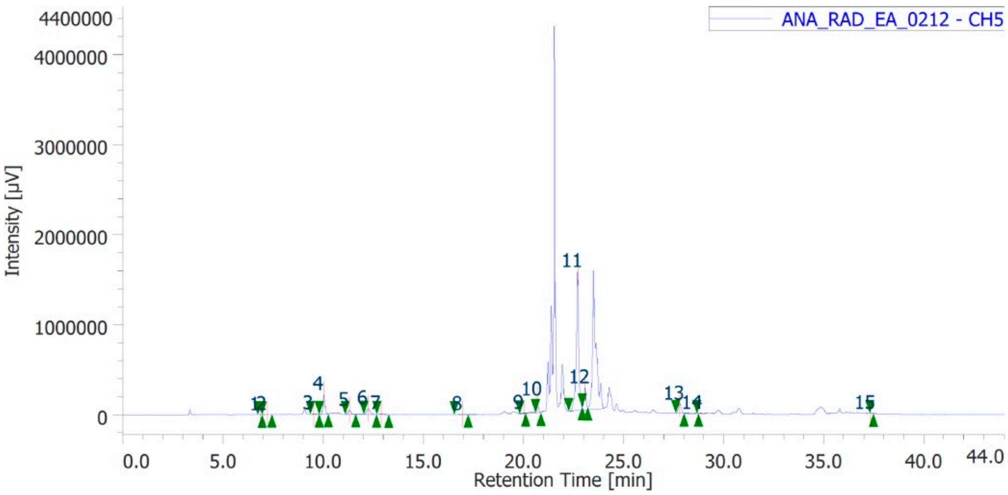

Figure S2. HPLC-DAD chromatograms of *Anacyclus maroccanus* Ball (AM) and *Anacyclus radiatus* Loisel (AR) extracts. (a) AM methanolic extract (b) AM ethyl acetate extract (c) AR methanolic extract (d) AR ethyl acetate extract.

(a)

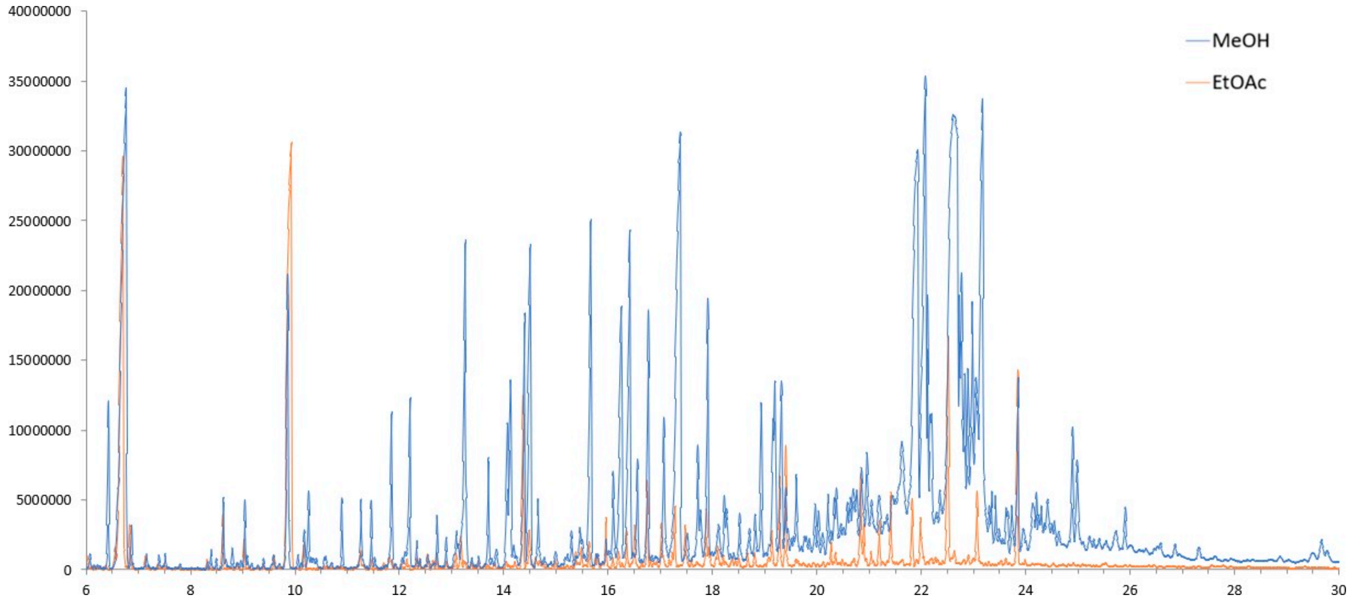

(b)

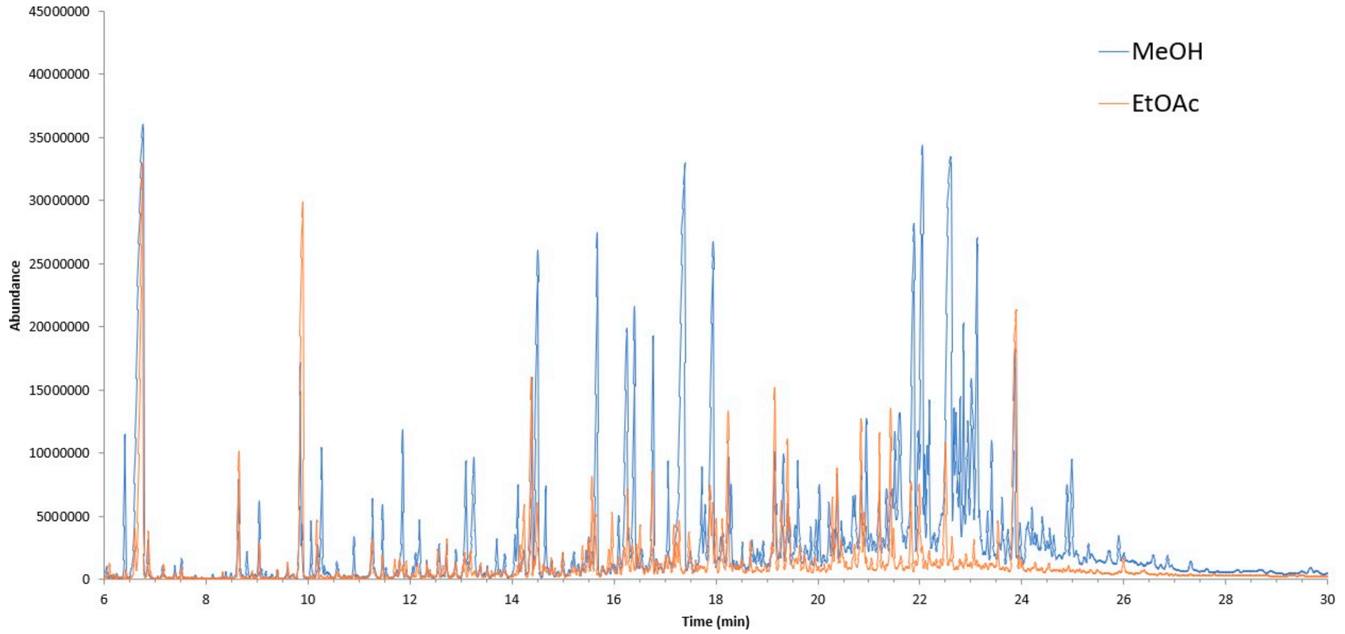

Figure S3. GC-MS chromatograms of *Anacyclus maroccanus* Ball (AM) and *Anacyclus radiatus* Loisel (AR) extracts. (a) AM methanolic and ethyl acetate extracts (b) AR methanolic and ethyl acetate extracts.

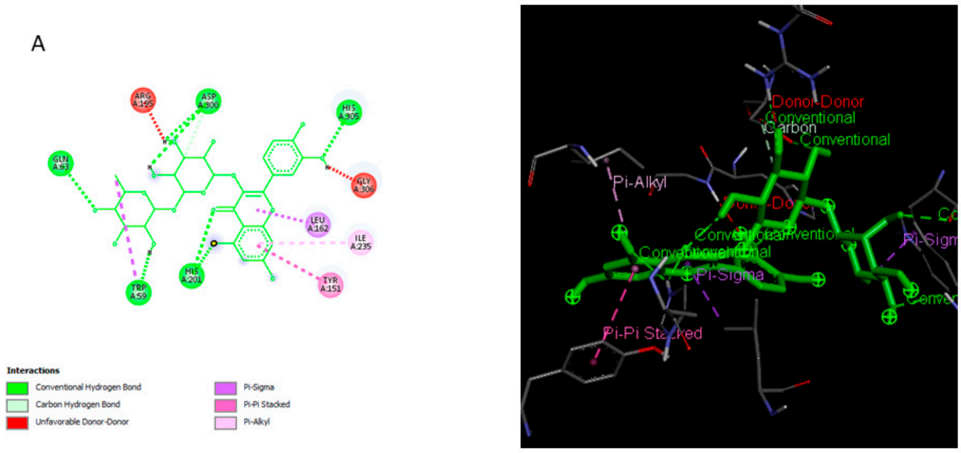

	Name	Visible	Color	Parent	Distance	Category	Types	From	From Chemistry	To	To Chemistry
1	:UNK0:H35 - :UNK0:O42	Yes		Ligand Non-bond Monitor	2,16478	Hydrogen Bond	Conventional Hydrogen Bond	:UNK0:H35	H-Donor	:UNK0:O42	H-Acceptor
2	:UNK0:H36 - A:ASP300:OD2	Yes		Ligand Non-bond Monitor	3,02701	Hydrogen Bond	Conventional Hydrogen Bond	:UNK0:H36	H-Donor	A:ASP300:OD2	H-Acceptor
3	:UNK0:H41 - A:TRP59:O	Yes		Ligand Non-bond Monitor	2,74242	Hydrogen Bond	Conventional Hydrogen Bond	:UNK0:H41	H-Donor	A:TRP59:O	H-Acceptor
4	:UNK0:H37 - A:ASP300:OD1	Yes		Ligand Non-bond Monitor	2,51999	Hydrogen Bond	Conventional Hydrogen Bond	:UNK0:H37	H-Donor	A:ASP300:OD1	H-Acceptor
5	A:GLN63:HE22 - :UNK0:O39	Yes		Ligand Non-bond Monitor	2,52503	Hydrogen Bond	Conventional Hydrogen Bond	A:GLN63:HE22	H-Donor	:UNK0:O39	H-Acceptor
6	A:HIS201:HE2 - :UNK0:O42	Yes		Ligand Non-bond Monitor	1,97979	Hydrogen Bond	Conventional Hydrogen Bond	A:HIS201:HE2	H-Donor	:UNK0:O42	H-Acceptor
7	A:HIS201:HE2 - :UNK0:O31	Yes		Ligand Non-bond Monitor	2,3439	Hydrogen Bond	Conventional Hydrogen Bond	A:HIS201:HE2	H-Donor	:UNK0:O31	H-Acceptor
8	A:HIS305:HD1 - :UNK0:O34	Yes		Ligand Non-bond Monitor	2,49034	Hydrogen Bond	Conventional Hydrogen Bond	A:HIS305:HD1	H-Donor	:UNK0:O34	H-Acceptor
9	:UNK0:C20 - A:ASP300:OD2	Yes		Ligand Non-bond Monitor	3,2683	Hydrogen Bond	Carbon Hydrogen Bond	:UNK0:C20	H-Donor	A:ASP300:OD2	H-Acceptor
10	:UNK0:C38 - A:TRP59	Yes		Ligand Non-bond Monitor	3,82702	Hydrophobic	Pi-Sigma	:UNK0:C38	C-H	A:TRP59	Pi-Orbitals
11	A:LEU162:CD1 - :UNK0	Yes		Ligand Non-bond Monitor	3,97081	Hydrophobic	Pi-Sigma	A:LEU162:CD1	C-H	:UNK0	Pi-Orbitals
12	:UNK0 - A:TYR151	Yes		Ligand Non-bond Monitor	4,74603	Hydrophobic	Pi-Pi Stacked	:UNK0	Pi-Orbitals	A:TYR151	Pi-Orbitals
13	:UNK0 - A:ILE235	Yes		Ligand Non-bond Monitor	5,19515	Hydrophobic	Pi-Alkyl	:UNK0	Pi-Orbitals	A:ILE235	Alkyl

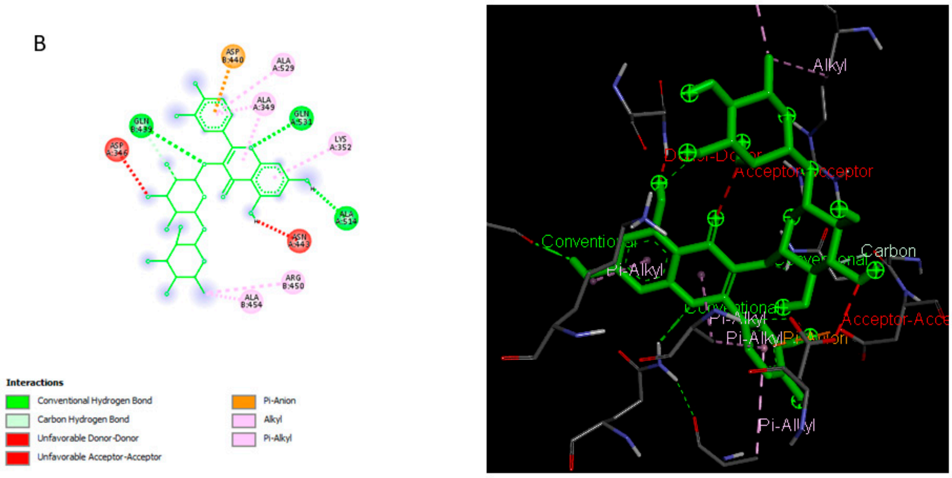

	Name	Visible	Color	Parent	Distance	Category	Types	From	From Chemistry	To	To Chemistry
1	:UNK0:H32 - A:ALA514:O	Yes		Ligand Non-bond Monitor	2,01488	Hydrogen Bond	Conventional Hydrogen Bond	:UNK0:H32	H-Donor	A:ALA514:O	H-Acceptor
2	A:GLN531:HE21 - :UNK0:O9	Yes		Ligand Non-bond Monitor	2,3651	Hydrogen Bond	Conventional Hydrogen Bond	A:GLN531:HE21	H-Donor	:UNK0:O9	H-Acceptor
3	B:GLN439:HE22 - :UNK0:O17	Yes		Ligand Non-bond Monitor	2,51056	Hydrogen Bond	Conventional Hydrogen Bond	B:GLN439:HE22	H-Donor	:UNK0:O17	H-Acceptor
4	:UNK0:C19 - B:GLN439:O	Yes		Ligand Non-bond Monitor	3,7218	Hydrogen Bond	Carbon Hydrogen Bond	:UNK0:C19	H-Donor	B:GLN439:O	H-Acceptor
5	B:ASP440:OD1 - :UNK0	Yes		Ligand Non-bond Monitor	3,49009	Electrostatic	Pi-Anion	B:ASP440:OD1	Negative	:UNK0	Pi-Orbitals
6	:UNK0:C38 - B:ARG450	Yes		Ligand Non-bond Monitor	4,71524	Hydrophobic	Alkyl	:UNK0:C38	Alkyl	B:ARG450	Alkyl
7	:UNK0:C38 - B:ALA454	Yes		Ligand Non-bond Monitor	4,00096	Hydrophobic	Alkyl	:UNK0:C38	Alkyl	B:ALA454	Alkyl
8	:UNK0 - A:ALA349	Yes		Ligand Non-bond Monitor	4,43425	Hydrophobic	Pi-Alkyl	:UNK0	Pi-Orbitals	A:ALA349	Alkyl
9	:UNK0 - A:LYS352	Yes		Ligand Non-bond Monitor	4,97635	Hydrophobic	Pi-Alkyl	:UNK0	Pi-Orbitals	A:LYS352	Alkyl
10	:UNK0 - A:ALA349	Yes		Ligand Non-bond Monitor	5,0339	Hydrophobic	Pi-Alkyl	:UNK0	Pi-Orbitals	A:ALA349	Alkyl
11	:UNK0 - A:ALA529	Yes		Ligand Non-bond Monitor	4,75927	Hydrophobic	Pi-Alkyl	:UNK0	Pi-Orbitals	A:ALA529	Alkyl

Figure S4. (A) Putative interactions between rutin and α-amylase (PDB: 1B2Y). Free energy of binding (ΔG) and affinity (Ki) are −9.3 kcal/mol and 180 nM, respectively. (B) Putative interactions between rutin and α-glucosidase (PDB: 1B2Y). Free energy of binding (ΔG) and affinity (Ki) are −10.3 kcal/mol and 29 nM, respectively.
